# Autologous patient-derived exhausted nano T-cells exploit tumor immune evasion to engage an effective cancer therapy

**DOI:** 10.1186/s12943-024-01997-x

**Published:** 2024-05-09

**Authors:** José L. Blaya-Cánovas, Carmen Griñán-Lisón, Isabel Blancas, Juan A. Marchal, César Ramírez-Tortosa, Araceli López-Tejada, Karim Benabdellah, Marina Cortijo-Gutiérrez, M. Victoria Cano-Cortés, Pablo Graván, Saúl A. Navarro-Marchal, Jaime Gómez-Morales, Violeta Delgado-Almenta, Jesús Calahorra, María Agudo-Lera, Amaia Sagarzazu, Carlos J. Rodríguez-González, Tania Gallart-Aragón, Christina Eich, Rosario M. Sánchez-Martín, Sergio Granados-Principal

**Affiliations:** 1 UGC de Oncología Médica, Hospital Universitario de Jaén, Jaén, 23007 Spain; 2 Instituto de Investigación Biosanitaria ibs.GRANADA, University Hospitals of Granada- University of Granada, Granada, 18100 Spain; 3 Centre for Genomics and Oncological Research, GENYO, Pfizer/University of Granada/Andalusian Regional Government, Granada, 18016 Spain; 4 Department of Biochemistry and Molecular Biology 2, Faculty of Pharmacy, University of Granada, Campus de Cartuja s/n, Granada, 18071 Spain; 5 Excellence Research Unit “Modeling Nature” (MNat), University of Granada, Granada, 18100 Spain; 6 UGC de Oncología, Hospital Universitario San Cecilio, Granada, 18016 Spain; 7 Department of Medicine, University of Granada, Granada, 18016 Spain; 8 Biopathology and Regenerative Medicine Institute (IBIMER), Centre for Biomedical Research, (CIBM), University of Granada, Granada, 18100 Spain; 9 Department of Human Anatomy and Embryology, Faculty of Medicine, University of Granada, Granada, 18016 Spain; 10 UGC de Anatomía Patológica, Hospital San Cecilio, Granada, 18016 Spain; 11 Department of Medicinal & Organic Chemistry and Excellence Research Unit of “Chemistry Applied to Biomedicine and the Environment”, Faculty of Pharmacy, University of Granada, Campus de Cartuja s/n, Granada, 18071 Spain; 12 Department of Applied Physics, Faculty of Science, University of Granada, Granada, 18071 Spain; 13 Laboratorio de Estudios Cristalográficos IACT-CSIC-UGR, Armilla, 18100 Spain; 14 UGC de Cirugía General y del Aparato Digestivo, Hospital Universitario San Cecilio, Granada, 18016 Spain; 15 Translational Nanobiomaterials and Imaging, Department of Radiology, Leiden University Medical Center, Leiden, 2333 The Netherlands

**Keywords:** Biomimetic nanoparticles, Immune evasion, PD1, PDL1, T-cell exhaustion, Immune checkpoint, Triple-negative breast cancer, Patient-derived xenograft, Immunotherapy

## Abstract

**Background:**

Active targeting by surface-modified nanoplatforms enables a more precise and elevated accumulation of nanoparticles within the tumor, thereby enhancing drug delivery and efficacy for a successful cancer treatment. However, surface functionalization involves complex procedures that increase costs and timelines, presenting challenges for clinical implementation. Biomimetic nanoparticles (BNPs) have emerged as unique drug delivery platforms that overcome the limitations of actively targeted nanoparticles. Nevertheless, BNPs coated with unmodified cells show reduced functionalities such as specific tumor targeting, decreasing the therapeutic efficacy. Those challenges can be overcome by engineering non-patient-derived cells for BNP coating, but these are complex and cost-effective approaches that hinder their wider clinical application. Here we present an immune-driven strategy to improve nanotherapeutic delivery to tumors. Our unique perspective harnesses T-cell exhaustion and tumor immune evasion to develop a groundbreaking new class of BNPs crafted from exhausted T-cells (NExT) of triple-negative breast cancer (TNBC) patients by specific culture methods without sophisticated engineering.

**Methods:**

NExT were generated by coating PLGA (poly(lactic-co-glycolic acid)) nanoparticles with TNBC-derived T-cells exhausted in vitro by acute activation. Physicochemical characterization of NExT was made by dynamic light scattering, electrophoretic light scattering and transmission electron microscopy, and preservation and orientation of immune checkpoint receptors by flow cytometry. The efficacy of chemotherapy-loaded NExT was assessed in TNBC cell lines in vitro. In vivo toxicity was made in CD1 mice. Biodistribution and therapeutic activity of NExT were determined in cell-line- and autologous patient-derived xenografts in immunodeficient mice.

**Results:**

We report a cost-effective approach with a good performance that provides NExT naturally endowed with immune checkpoint receptors (PD1, LAG3, TIM3), augmenting specific tumor targeting by engaging cognate ligands, enhancing the therapeutic efficacy of chemotherapy, and disrupting the PD1/PDL1 axis in an immunotherapy-like way. Autologous patient-derived NExT revealed exceptional intratumor accumulation, heightened chemotherapeutic index and efficiency, and targeted the tumor stroma in a PDL1^+^ patient-derived xenograft model of triple-negative breast cancer.

**Conclusions:**

These advantages underline the potential of autologous patient-derived NExT to revolutionize tailored adoptive cancer nanotherapy and chemoimmunotherapy, which endorses their widespread clinical application of autologous patient-derived NExT.

**Supplementary Information:**

The online version contains supplementary material available at 10.1186/s12943-024-01997-x.

## Background

Nanoparticle-based nanomedicine has significantly improved drug delivery by targeting, passively accumulating within tumor tissues, and effectively penetrating cancer cells. Nanoparticles (NPs) present augmented attributes such as enhanced bioavailability, improved biodistribution, increased solubility, prolonged retention, and controlled release of therapeutic payloads, thereby reducing systemic toxicity and mitigating the adverse effects associated with chemotherapy [1]. Various mechanisms result in uneven accumulation of NPs among patients, thus adversely impacting the antitumor effectiveness of chemotherapy. Hence, attaining a more precise and elevated intratumor accumulation of NPs through active targeting is pivotal for optimizing drug delivery and efficacy. This strategy hinges on the utilization of NPs possessing surface modifications with ligands or receptors that exhibit specificity towards corresponding molecules expressed at the tumor site, such as EGFR or PDL1, among others [2]. Nonetheless, surface functionalization entails intricate procedures that escalate costs and timelines, posing challenges for its clinical application [2, 3].

Several of those limitations are addressed by biomimetic NPs (BNPs), which are nanocarriers coated with membranes derived from diverse cell types including erythrocytes [4], macrophages [5], T-cells [6, 7], CAR-T cells [8], NK [9], dendritic cells [10], mesenchymal stem cells [11], cancer cells [12], hybrids [13], or platelets [14]. BNPs retain the surface molecular diversity and functionalities, thereby conferring superior biocompatibility, reduced immunogenicity, evasion of immune system clearance, prolonged circulation, enhanced passive accumulation and penetration within the tumor site, and specific active tumor targeting. Consequently, BNPs hold immense promise for clinical applications in cancer therapy [15–17]. Their avidity and specificity towards diseased tissues can be fine-tuned by the appropriate selection of the source cell type. For instance, T-cells represent a promising option for coating NPs due to their inherent tumor affinity and recognition of specific antigens on cancer cells [15]. Although some studies have cloaked NPs with T-cell membranes, they rely on TCR-based single targeting [6, 7], which can jeopardize their antitumor efficacy [15]. Given that unmodified cells might limit BNP functions, engineered-cell-derived alternatives have been developed to enhance their capabilities. These modifications include enabling single targeting against specific antigens [18], achieving dual targeting on engineered tumor cells in vitro [19], or enhancing immunotherapy (IT) [20, 21]. However, integrating these complex and potentially costly methods into hospitals worldwide presents considerable challenges.

In this study, we refrain from relying on sophisticated engineering methods to procure modified cells exhibiting improved functionalities. Rather, drawing inspiration from adoptive T-cell therapies, we emulate the mechanism of tumor immune evasion (IE), where cancer cells crosstalk with T-cells through different ligand-receptor interactions, introducing a new class of BNPs designed for active drug delivery. IE is a hallmark of cancer cells to avoid host antitumor immunity that facilitates tumor growth and progression. These cells evade immunity through different mechanisms such as immune cell deactivation through the surface expression or secretion of inhibitory ligands (e.g., PDL1, Galectin-3, FGL-1, MHCII, Galectin-9, HMGB1, Ceacam-1) that bind to their corresponding immune checkpoint (IC) receptors (e.g., PD1, LAG3, TIM3) on the surface of immune cells. Furthermore, cancer cells can establish an immunosuppressive tumor microenvironment (TME) that induces T-cell exhaustion, where these cells become hypofunctional and overexpress inhibitory checkpoint receptors [22–25]. Therefore, while IE is a characteristic strength of cancer cells, we leverage it as a vulnerability to effectively administer chemotherapy to tumor cells PLGA (poly(lactic-co-glycolic acid) NPs, approved by the FDA and EMA for drug delivery in humans [26], coated with membranes of exhausted T-lymphocytes (NExT) obtained from cancer patients.

**Fig. 1:**
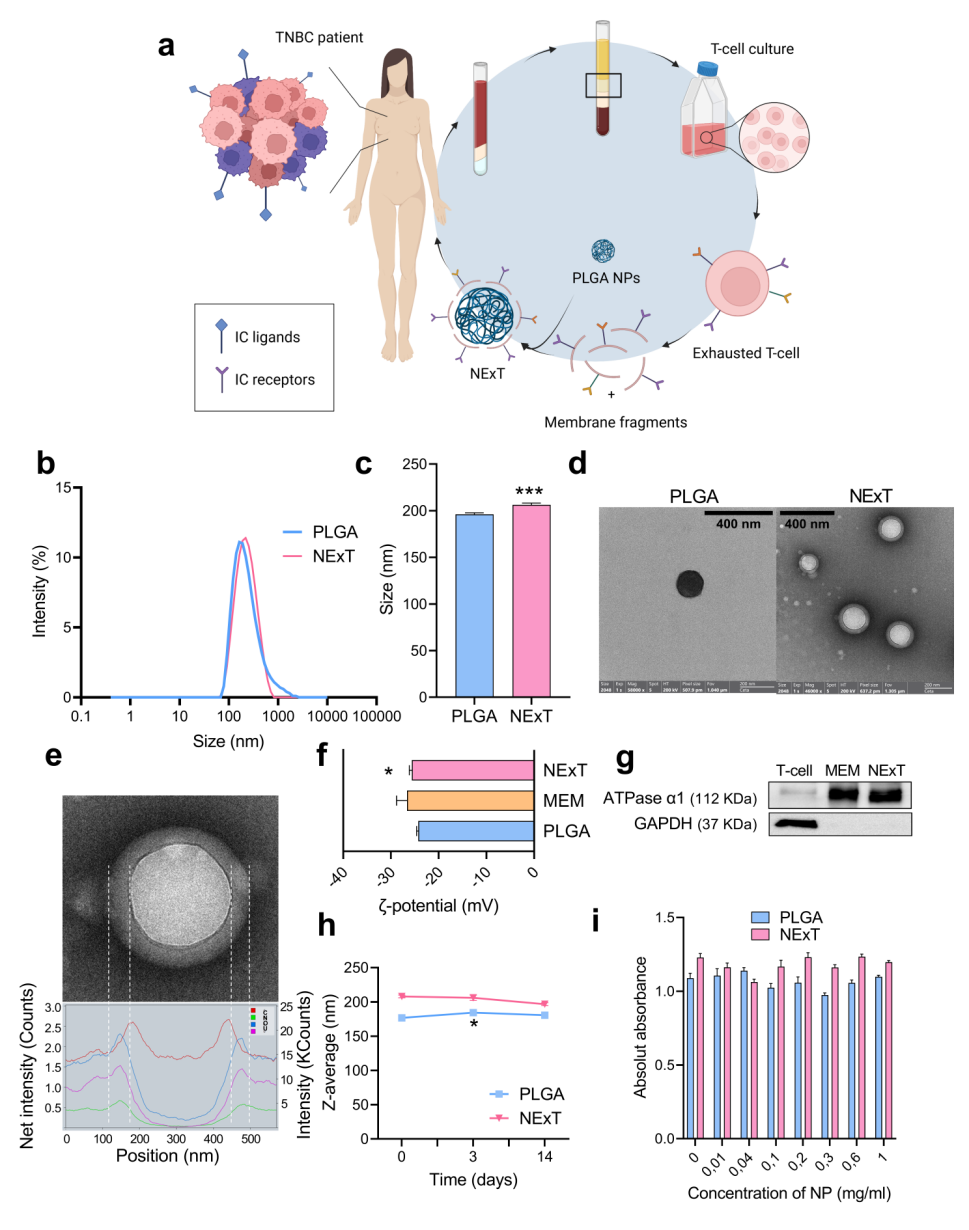
Synthesis and characterization of biomimetic nanoparticles. **a** Schematic illustration of the synthesis process of biomimetic nanoparticles coated with membranes of exhausted T-lymphocytes (NExT) from TNBC. **b** Size distribution of PLGA and NExT nanoparticles. **c** Average size of PLGA and NExT nanoparticles (*n* = 1 patient). Comparison with PLGA: ***p < 0.001. **d** TEM images of PLGA and NExT particles. **e** Chemical composition profile of NExT by TEM. **f** Zeta-potential of PLGA, membranes, and NExT (*n* = 1 patient) in MilliQ water (pH 6.6–6.8). Comparison with PLGA: *p < 0.05. **g** Western blot of the α1 subunit of ATPase (ATPase α1), and GAPDH in T-cells, T-cell membranes, and NExT. **h** Size (z-average) showing the stability of PLGA and NExT for 14 days in PBS (*n* = 1 patient). Comparison with day 0: *p < 0.05. **i** Toxicity of PLGA and NExT in SUM159 at different concentrations (*n* = 1 patient, six replicates). Data are represented as mean ± SEM. *p < 0.05, and ***p < 0.001

Our hypothesis posits that NExT will retain several IC receptors, enabling the interaction with their corresponding ligands on tumor cells that are licensed to evade antitumor immunity. This approach aims to bolster the specificity and intratumor accumulation of NPs, ultimately enhancing the therapeutic index of chemotherapy. To validate our hypothesis, we focused on triple-negative breast cancer (TNBC), recognized as an immunogenic breast cancer subtype due to its higher mutational burden, percentage of tumor-infiltrating lymphocytes, and PDL1 expression, which ultimately support the application of IT [27]. The present work will investigate whether NExT can improve the therapeutic profile of drugs that represent the standard of care for unresectable TNBC (the taxane docetaxel and the anthracyclines doxorubicin and epirubicin) [28]. Similar to adoptive T-cell therapy, when NExT are autologously administered to patients, as an adoptive nanotherapy, they will become a highly precise and effective delivery system for a wide range of therapeutic agents, providing a potential avenue for treating TNBC and other tumors capable of evading the immune system (Fig. 1a).

## Methods

### Patient samples

The study recruited 24 patients diagnosed with TNBC from the Oncology CMU of the Hospital Clinico San Cecilio in Granada, Spain. Peripheral blood samples (8 ml) and one-patient core needle tumor biopsy were collected according to the protocol approved by the Reference Ethics Committee with code PI19/01533/1626-N-19.

### Cell cultures and PDL1 expression on tumor cells

Human breast tumor cells MCF7, MDA-MB-231, MDA-MB-468, BT549, and Hs578T from ATCC, and SUM159 (TNBC) from Asterand, were cultured in DMEM (Dulbecco’s Modified Eagle Medium, Sigma-Aldrich), supplemented with 10% FBS (fetal bovine serum, Thermo Fisher Scientific), at 37 °C and 5% CO_2_. Namalwa (CRL-1432) and Nalm7 (CRL-3273) cells from ATCC were cultured in RPMI-1640 media supplemented with 10% FBS and 100 U/mL penicillin/streptomycin (1%P/S, Biowest) at 37 °C and 5% CO_2_. Namalwa and Nalm7 cells were transduced with lentiviral vectors to achieve a final expression of PDL1 at a multiplicity of infection (MOI) 20 through spinoculation (800×g for 30 min at 32 °C).

PDL1 expression was induced in tumor cells by treatment with IFNγ (100 ng/ml) for 24 h [29]. PDL1 levels were determined by flow cytometry by incubating cancer cells with anti-CD274 (5 µg/ml, PDL1, MIH1, PE; eBioscience), or isotype (5 µg/ml, Mouse IgG1 kappa Isotype, PE; eBioscience) for 15 min at room temperature. The percentage of positive cells for each antibody was determined with a FACSVerse flow cytometer (BD Biosciences). Analysis of the results was performed using FlowJo.

### Culture, activation, and expansion of PBMCs

PBMCs were isolated from peripheral blood in BD Vacutainer CPT – Sodium Heparin tubes (BD Biosciences) by centrifugation at 1,800×g for 30 min at room temperature. PBMCs were washed twice with 1X PBS supplemented with FBS (2%) (wash buffer) at 450×g for 10 min at room temperature. Culture and activation of PBMCs to obtain T-lymphocyte-enriched cultures without magnetic selection were performed as previously published, with modifications [30, 31]. Briefly, cells were transferred to a 12-well Nunclon flat-bottom plate (Thermo Fisher Scientific) (4–8 million cells/well) with 2 ml of RPMI-1640 medium (Sigma-Aldrich) supplemented with 10% human AB serum (Sigma-Aldrich), penicillin-streptomycin antibiotic (1%) (Gibco), and IL-2 (100 U/ml) (Peprotech) (RPMI+) and incubated at 37 °C and 5% CO2 for 4 days without manipulation. After these 4 days of acclimatization without manipulation, suspension cells were isolated from adherent cells and expanded in fresh RPMI + medium with T Cell TransAct (10 µl/ml) for 3 weeks. The RPMI + culture medium was renewed every 48–72 h. The evolution of proliferation was checked by manual counting with trypan blue (Gibco). The percentage of T (CD3+) and B (CD19+) cells was investigated in T-cell-enriched cultures expanded for 3 weeks and incubated with anti-CD3 (2.5 µg/ml, anti-human CD3, OKT3, PerCP Cyanine 5.5; eBioscience) and anti-CD19 (10 µg/ml, anti-human CD19, SJ25C1, APC; eBioscience) for 15 min at room temperature. The percentage of positive cells for each antibody was determined with a FACSVerse flow cytometer (BD Biosciences) and analyzed with FlowJo.

### Acute induction of exhausted T-cellsin vitro

Exhausted T-lymphocytes (ExT) were obtained by acute activation of T-cell-enriched cultures with T Cell TransAct (10 µl/ml) for 24, 48, and 72 h before collection. Expression of the inhibitory IC receptors PD1, LAG3, TIM3, and TIGIT was determined by flow cytometry in ExT after incubation with anti-PD1 (5 µg/ml, anti-human CD279, J105, PE; eBioscience), anti-LAG3 (0.3 µg/ml, anti-human CD223, 3DS223H, PE or APC; eBioscience), anti-TIGIT (0.625 µg/ml, anti-human TIGIT, MBSA43, FITC; eBioscience), anti-TIM3 (0.625 µg/ml anti-human CD366, F38-2E2, PE or APC; eBioscience), or the corresponding isotype (Mouse IgG1 kappa Isotype Control, PE, APC or FITC; eBioscience), for 15 min at room temperature.

### Isolation of membranes

Isolation of cell membranes was performed using a modified protocol based on previous publications [32]. For this purpose, T-cell-enriched cultures (NaT) or ExT were washed with 1X PBS and resuspended in a ratio of 3 million cells per ml of hypotonic Tris-Magnesium buffer (Tris 10 mM, MgCl_2_ 1 mM, pH 7.4), supplemented with 1X protease and phosphatase inhibitor (Thermo Fisher Scientific), EDTA (0.5 mM) (Thermo Fisher Scientific) and benzonase (12.5 U/ml) (Santa Cruz Biotechnology), at 4 °C for 20 min. Cells were homogenized for 40 s with an Ika Ultra-Turrax T18 disperser at 11,000 rpm and centrifuged at 600×g for 10 min at 4 °C to discard larger cell debris. The supernatant was centrifuged at 17,000×g for 30 min at 4 °C. The protein content of the resulting pellet was quantified using the Pierce BCA Protein Assay kit (Thermo Fisher Scientific). The resulting membranes were resuspended in MilliQ water at a concentration of 1–2 mg protein/ml.

### Synthesis of PLGA NPs

PLGA NPs were synthesized by a double emulsion solvent evaporation (W/O/W) technique as previously published, with modifications [33]. For this purpose, 25 mg of PLGA (acid terminated, lactide: glycolide 50:50, Mw 24–38 kDa) (Resomer RG 503 H. Sigma-Aldrich) was dissolved in 1.5 ml dichloromethane (DCM) alone (empty PLGA) or with coumarin-6 (Sigma-Aldrich) (37.5 µg), IR780 (1 mg) (Sigma-Aldrich), epirubicin (EPI) (2 mg) or doxorubicin (DOX) (2 mg) (MedChem Express). This organic solution was emulsified dropwise with 0.5 ml of polyvinyl alcohol (PVA) (0.001%) with a probe sonicator (100 W, 100% amplitude) for 3 min (UP100H, Hielscher). The obtained emulsion (W/O) was added dropwise to 24.5 ml of PVA (0.001%) and sonicated again (100 W, 100% amplitude) for 3 min to form a double emulsion (W/O/W). The DCM was evaporated by magnetic stirring for 2 h. For docetaxel (DOC) encapsulation, the above protocol was modified according to Cho et al. [34]. For this purpose, 6 mg of docetaxel (MedChem Express) was dissolved in 0.5 ml of DCM and subsequently, 25 mg of PLGA was added. Both solutions were mixed and sonicated (100 W, 100% amplitude) for 2 min. The obtained mixture was added to 10 ml of PVA (0.2%) and sonicated again (100 W, 100% amplitude) for 10 min. The DCM was evaporated by magnetic stirring for 2–3 h.

### Preparation of coated BNPs

Coating of PLGA NPs, empty or loaded with a drug or imaging agent, was performed by sonication as published [35]. First, to optimize the coating efficiency, cell membranes were labeled with DiL (Tetramethylindocarbocyanine Perchlorate 2.5 µM; Invitrogen) by incubation for 20 min at 37 °C, and the excess of the fluorophore was washed by centrifugation at 17,000×g for 30 min at 4 °C. PLGA NPs (1 mg/ml), were sonicated for 5, 10, and 15 min in a bath sonicator at 100 W and 40 kHz (GT SONIC-R3) with different concentrations of DiL-labeled membrane (1 and 2 mg protein/ml) at PLGA: membrane protein ratios 1:1 and 1:2. For this purpose, FITC+ (Nanovex Biotechnologies) and FITC– PLGA NPs decorated with DiL-labeled membranes were washed at 9600×g for 5 min at 4 °C, the pellet was resuspended in 1X PBS and analyzed on a FACSVerse cytometer (BD Biosciences). Results were analyzed with FlowJo.

The optimized coating protocol to prepare NExT and NPs coated with membranes from T-cell enriched cultures (NNaT) was established as follows: PLGA NPs (1 mg/ml) and the membrane fraction (2 mg protein/ml) of ExT or NaT were mixed in a 1:1 volume ratio (core: membrane), and sonicated for 5 min in a bath sonicator at 100 W and 40 kHz.

### Characterization of BNPs

The size, PDI (polydispersity index), and ζ-potential in MilliQ water (pH 6.6–6.8) of PLGA NPs, NExT, and NNaT were characterized in a Zetasizer Nano S system (Malvern Instrument, UK) by DLS (dynamic light scattering) and ELS (electrophoretic light scattering). The self-optimization routine in the Zetasizer software was used for all measurements, and the ζ-potential was calculated according to the Smoluchowsky theory. Transmission Electron Microscopy (TEM) as previously published [12]. Briefly, 50 µl of NPs were left on a carbon-coated 300 square mesh copper grid for 5 min. Negative staining of samples (PLGA and NExT) was made with 1% uranyl acetate (Sigma Aldrich) after washing with water for 1 min. After drying the samples with paper at room temperature, their chemical composition and imaging were made with a Thermo Fisher TALOS F200X high-resolution transmission microscope at 200 kV. For stability assay, PLGA and NExT were conserved at 4 °C for 0, 3, and 14 days and their z-average (size) was measured by DLS.

### Preservation and orientation of membrane proteins

The presence of membrane proteins on the surface of BNPs was determined by Western blotting as published [13]. Membrane-coated PLGA NPs were purified by centrifugation at 9600×g for 10 min. Whole T-cell-enriched culture extract, membrane fraction, and membranes isolated from coated PLGA NPs by centrifugation at 9,600×g for 10 min (30 µg protein) were subjected to electrophoresis and transferred onto a nitrocellulose membrane. Membranes were incubated overnight at 4 °C with primary antibodies against GAPDH (1E6D9, Proteintech) or Na^+^/K^+^-ATPase α1 (C464.6, Santa Cruz Biotechnology) (1:1,000 dilution), and the secondary antibody (Cell Signaling) (1:2,000 dilution) for 1 h at room temperature. The chemiluminescence signal was obtained with an ImageQuant LAS 4000 (GE Healthcare).

The surface expression of IC receptors on NExT was determined by flow cytometry (FACSVerse flow cytometer, BD Biosciences) with no threshold on the forward scatter to detect the nanoparticles as previously reported [6, 36]. Briefly, NExT were incubated for 15 min at room temperature with anti-PD1, anti-LAG3, and anti-TIM3 antibodies, or their corresponding isotype, as described above. The results were analyzed by FlowJo.

### Specific targeting of tumor cells by NExT

SUM159 cells were treated with NExT or PLGA NPs (100 µg/ml) loaded with coumarin-6 for 5, 15, and 30 min. The MDA-MB-468 cell line was cultured in the presence/absence of IFNγ (100 ng/ml) for 24 h and treated with NExT or NNaT (100 µg/ml) loaded with coumarin-6. For the rescue experiment, MDA-MB-468 cells (with/without IFNγ, 100 ng/ml) were treated with the anti-PDL1 antibody atezolizumab (10 µg/ml) for 24 h and coumarin-6-loaded NExT or PLGA NPs (100 µg/ml) for 15 min. PDL1-transduced or wild-type Namalwa and Nalm7 cells were treated with NExT or PLGA NPs (100 µg/ml) loaded with coumarin-6 for 5 and 15 min, and 30 and 60 min, respectively. The percentage of cells positive for coumarin-6 fluorescence was detected in the FITC channel by the FACS Verse cytometer and analyzed with FlowJo.

### Encapsulation efficiency and drug release

To determine the mass of the drug encapsulated, chemotherapy-loaded NPs were dissolved in dimethyl sulfoxide (DMSO) (Sigma-Aldrich). DOC concentration was measured by HPLC using a calibration curve in the concentration range from 0.00 to 500.0 µg/ml (R^2^ = 0.9998). The concentration of DOX and EPI was measured spectrophotometrically at 480 nm using a calibration curve in the concentration range of 0.00 to 100.0 µg/ml (R^2^ = 0.9992) in an Infinite 200 PRO plate reader.

The encapsulation efficiency (EE) of each drug was calculated according to Eq. 1:

1 $${\rm{EE}}\,({\rm{\% ) = }}\,{{mass\,of\,drug\,encapsulated} \over {total\,mass\,of\,drug}}\, \times \,{\rm{100}}$$

The loading capacity (LC) of each drug was calculated according to Eq. ;2:

2 $$\eqalign{{\rm{LC}}\,({\rm{\% ) = }} & \,{{mass\,of\,drug\,encapsulated} \over {mass\,of\,drug\,encapsulated + \,mass\,of\,NPs}}\, \cr & \times \,{\rm{100}} \cr}$$

The release profile of each drug from NExT and PLGA NPs was determined by measuring the concentration of free drug in an aqueous receptor phase over 4 weeks as previously reported [37, 38]. Briefly, NPs (2 mg) were washed and resuspended in 200 µl of PBS Tween (Sigma-Aldrich) (0.1%) (pH 7.4) and incubated at 37 °C in an orbital shaker. Samples were collected at 1, 3, 6, 24, and 48 h, as well as at 1, and 2 weeks, centrifuged at 9,600×g for 5 min, and the supernatant was collected for further drug quantification as described above.

### In vitro toxicity and therapeutic efficacy of NExT

The toxicity of PLGA NPs and NExT was studied in SUM159 cells treated with increasing concentrations of NPs (from 0.01 to 1 mg/ml). The proliferation of SUM159 and MDA-MB-468 cells was assayed with increasing concentrations of PLGA NPs or NExT loaded with DOC (0–50 nM), DOX (0-1.8 µM), and EPI (0-500 nM) for 48 h. After treatments, the WST-1 reagent (Sigma-Aldrich) was added and incubated at 37^o^C for 1 h. Absorbance was measured at 450 nm.

### Animal studies

Animal welfare and experimental procedures were carried out according to institutional (Research Ethics Committee of the University of Granada) and international (Council of the European Communities) standards. All procedures were approved by the Institutional Committee for the Animal Care and Use of the University of Granada (code of the approved protocol: 12/07/2019/127). All animals were housed and maintained at 20–24 °C, 50% relative humidity, and a 10:14 h light-dark cycle with food and water *ad libitum.*

### In vivo safety of NExT

Toxicity assays were performed on 6- to 8-week-old female CD1 mice (*n* = 3 mice/group). PBS was used as a vehicle. Mice were treated with empty PLGA NPs and NExT (25 or 100 mg/kg) at a volume of 100 µl through the tail vein. The animals were maintained under standard conditions and body weight, response to handling, behavior, appetence, and other clinical signs (lack of grooming, aggressiveness towards peers, stereotypies, piloerection, nasal and ocular discharge, arched back, convulsions, severe respiratory distress, severe dehydration, immobility, social isolation, and hypothermia) were evaluated until endpoint. After 7 days, blood was withdrawn by cardiac puncture (terminal procedure), and major organs were collected after euthanasia, as published [39]. After a macroscopic examination, the organs were sectioned and embedded in 4% paraformaldehyde (PFA) for further histopathological study by a pathologist with hematoxylin/eosin (H&E). Hematological parameters (white blood cells, red blood cells, hemoglobin, hematocrit, mean corpuscular volume, mean corpuscular hemoglobin, mean corpuscular hemoglobin concentration, platelets) and white blood cell count were analyzed by the School of Clinical Analysis of the University of Granada.

### Intratumor accumulation and biodistribution of NExT

Intratumor accumulation of NExT was assayed in female NOD SCID Gamma (NSG) mice orthotopically injected with SUM159 cells (3 × 10^6^) in the mammary fat pad (*n* = 6 mice/group). When tumors reached 150–170 mm^3^, mice were randomized into experimental groups, namely Vehicle, PLGA, and NExT. Each mouse received an injection of IR780-loaded PLGA and NExT (100 µl) at a concentration of 100 µg/ml through the tail vein. This concentration results in 0.5 mg/kg of fluorophore. Fluorescence was measured at 0, 1, 2, 4, 6, 8, and 24 h on the IVIS Spectrum In Vivo Imaging System (PerkinElmer) using the 745/800nm filter. After 24 h, fluorescence was also measured in hearts, lungs, livers, kidneys, spleens, and tumors ex vivo. The results were normalized to the background fluorescence of the vehicle group.

### In vivo therapeutic activity of NExT in a PDX model of TNBC

We generated a patient-derived xenograft (PDX) (UGR01) model from a core needle biopsy of a TNBC patient enrolled at the University Hospital San Cecilio (ibs.GRANADA) as we published [40]. Briefly, tumor biopsy (1 mm^3^) was orthotopically implanted into the cleared mammary fat pad of 4-to-5-week-old female NSG mice. PDX tumor tissue (G0) was excised and cut into small (1 mm^3^) fragments and then re-implanted in new mice (3–4 mice) to obtain G1. This process was repeated until G3 was generated. Early generations were fixed in 4% PFA and embedded in paraffin for further histopathological characterization by a pathologist. When G3 tumors reached 120–170 mm^3^ in size, the mice were randomly assigned to treatment groups (*n* = 5 mice/group): Vehicle (1X PBS), Free-DOC (5 mg/kg), PLGA-DOC (5 mg/kg), and autologous NExT-DOC (5 mg/kg) coated with membranes of T-cells derived from the source patient of tumor biopsy to generate the PDX model UGR01. Mice received 4 injections of 100 µl through the tail vein (cumulative dose of DOC: 20 mg/kg). Tumor growth was assessed twice weekly with a digital caliper and the tumor volume was calculated by Eq. ;3 as we published [40]. Finally, PDX mice were sacrificed, and tumors were fixed with 4% PFA for further analysis.

3 $$V\, = \,lengt{h^2}\, \times \,width\,\, \times \,{\pi \over 6}$$

### Immunofluorescence, immunohistochemistry, histochemistry and FISH

Livers, lungs, and PDX tumor tissue were fixed in 4% PFA at 4 °C for 24 h, washed in 0.1 M PBS, embedded in paraffin with an automatic tissue processor (TP1020; Leica, Germany), and cut in Sect. (4 μm). Immunofluorescence was performed as we published [41]. Briefly, sections were deparaffinized with xylene and hydrated with decreasing alcohol concentrations. For immunofluorescence, antigen retrieval was performed at 121 °C for 15 min in a sodium citrate buffer solution (pH 6.0). Then, sections were blocked for 2 h at room temperature with 5% BSA and incubated with the primary antibodies anti-PDL1 (CD274, MIH1, eBioscience) (1:100 dilution), anti-PDL1 (CD274, 2B11D11, Proteintech) (1:100 dilution), anti-Ki67 (8D5, Cell Signaling) (1:1000 dilution), and anti-α-SMA (α-Smooth muscle actin) (ab5694, Abcam) (1:100 dilution) overnight at 4 °C. Samples were washed thrice with PBS and incubated with the appropriate secondary antibody (anti-rabbit Alexa Fluor 488 or anti-mouse Alexa Fluor 594; Cell Signaling) (1:500 dilution) for 2 h at room temperature. Where indicated, cell membranes were stained with DiL (2.5 µM; Invitrogen) by incubation for 20 min at 37 °C. Finally, it was washed thrice with PBS and mounted with a DAPI-containing mounting medium (Cell Signaling). Images were taken with a confocal microscope Zeiss LSM 710.

Immunohistochemistry was performed after antigen retrieval (Antigen Retrieval fluid 10X EDTA, pH 8.0; Vitro) in a PTLink module (Vitro). Staining was made in an Autostainer 480 (Vitro) by using the Master Polymer Plus Detection System (Peroxidase) (Vitro). Briefly, sections were washed and blocked with 3% hydrogen peroxide for 5 minutes. The primary antibodies against ER (rabbit monoclonal antibody, clone SP1; Vitro) and PR (rabbit monoclonal antibody, clone 16; Vitro) were applied for 5 and 10 min at room temperature, respectively. Sections were then treated with immunodetection solution (biotinylated secondary antibody) for 30 min, and 3,3’-diaminobenzidine (1:50 dilution) (Vitro) as the chromogenic agent. Sections were counterstained in Meyer’s hematoxylin. As a negative control, the primary antibody was replaced by a non-immune serum. The absence of any nuclear staining in neoplastic cells was considered negative by a pathologist.

Deparaffinized sections of livers, lungs, and PDX tumor tissue were stained with H&E and further assessed by a pathologist. The sections were hydrated (deparaffinized), stained with Hematoxylin and Eosin (Sigma-Aldrich), and dehydrated according to the manufacturer’s instructions. The stained slides were mounted on coverslips with mounting medium. The images were obtained using a Leica DM 550B microscope.

HER2 status was determined using the fluorescence in situ hybridization (FISH) test in the deparaffinized sections of PDX tumor tissue (ERBB2/CCP17 FISH Probe Kit, CT-PAC001, CytoTest Inc). The latest ASCO/CAP 2018 recommendations for condition assessment were used for interpretation by a pathologist [42].

### Statistical analysis

Statistical differences between two experimental groups were analyzed using a Student’s t-test, and differences between groups were analyzed by one-way ANOVA with GraphPad Prism 5.0 (GraphPad Software Inc.). The experiments were conducted at least in triplicates unless otherwise specified. Results are shown as mean ± standard error of the mean (SEM). A *p*-value < 0.05 was considered statistically significant.

## Results

### NExT showed efficient coating and physicochemical features for enhanced drug encapsulation and cytotoxicity

Our results by DLS show that PLGA NPs had a mean diameter of 193.5 ± 5.38 nm (Fig. 1b, c), with a PDI of 0.205. To obtain BNPs, PLGA cores were decorated with membranes from T-cell-enriched cultures derived from PBMCs of TNBC patients (NExT). Our results showcased that the culture/expansion protocol generated T-cell-enriched cultures (∼ 90% of T-cells) with adequate cell numbers for the coating procedure (Fig. S1). Coating efficiency was optimized at concentrations of PLGA cores and purified membranes of 1 and 2 mg/ml, respectively, with a core: membrane volume ratio of 1:1 and sonication for 5 min (Fig. S2a-c). The successful coating was verified by colocalization of FITC+-PLGA and DiL-stained membranes (Fig. S2d). Physicochemical analysis revealed that NExT were significantly larger, with a mean size of 207.72 ± 3.33 nm, a mean PDI value of 0.236, and a range of diameter increase of 5–22 nm (Fig. 1c). TEM confirmed the successful coating, displaying typical core-shell structures of spherical shape, and size consistent with the hydrodynamic diameter measured by DLS (NExT: ∼ 210–220 nm; PLGA: ∼ 180–200 nm) (Fig. 1d). Chemical composition profiling by TEM showed the increased presence of N, O, C, and U around the core, which confirms the presence of biological material and supports the correct membrane coating around the polymeric core (Fig. 1e). The surface charge (ζ-potential) of PLGA NPs and NExT was − 24.3 mV and − 25.6 mV, respectively, akin to the surface charge of pure T-cell membrane fraction (-26.6 mV) (Fig. 1f). The successful surface cloaking and the purity of the extracted T-cell membranes were also confirmed via western blotting. Our results demonstrated the enrichment in the α1 subunit of the transmembrane Na^+^/K^+^-ATPase protein in NExT and pure T-cell membranes, compared to the T-cell lysate. As expected, we observed the expression of cytosolic GAPDH protein in the whole T-cell lysate, whereas it was not detected in NExT and pure membranes (Fig. 1g). Next, we analyzed the stability of the PLGA cores and NExT by DLS in PBS for 14 days. We found an increase in the size of the PLGA nanoparticles on day 3 compared with day 0, which was maintained until the end of the experiment (day 14). On the contrary, we did not find such an increase in the size of NExT throughout the experiment, suggesting the higher stability of NExT compared with the PLGA cores by DLS in PBS for 14 days (Fig. 1h). Finally, we assessed the antiproliferative effects of the PLGA cores and NExT in SUM159 cells and found their negligible cytotoxicity at the tested concentrations (Fig. 1i).

### Correct preservation and orientation of immune checkpoint receptors on the surface of NExT

**Fig. 2:**
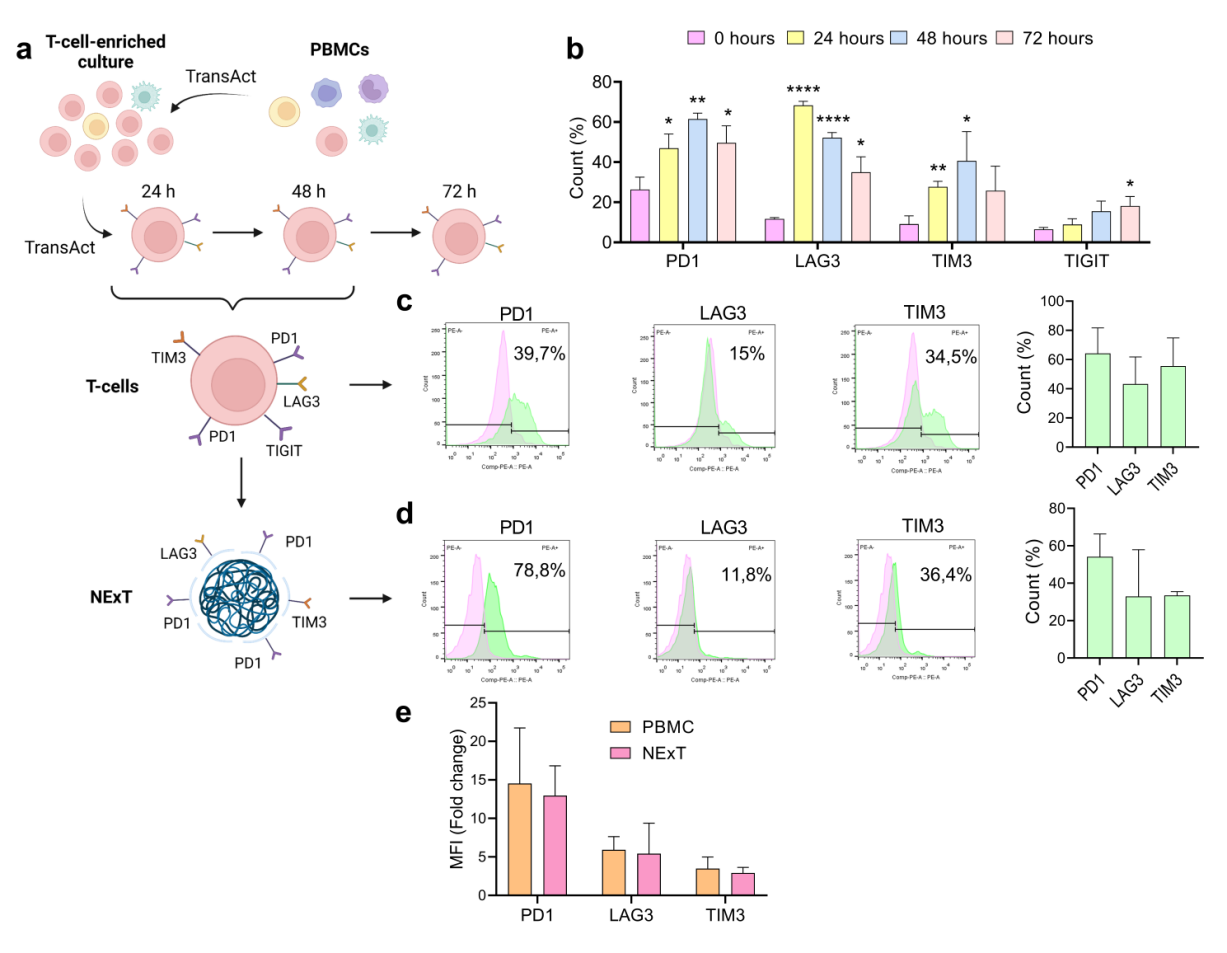
T-cell exhaustion and characterization of surface functionalization of NExT. **a** Schematic depicting the obtention of T-cell-enriched cultures, derived from TNBC patients, with high expression of immune checkpoint receptors for NExT preparation. **b** PD1, LAG3, TIM3, and TIGIT, and levels measured by flow cytometry in T-cell-enriched cultures after activation with TransAct at 0, 24, 48, or 72 h (*n* = 4 patients). **c** Representative flow cytometry histograms and PD1, LAG3, and TIM3 levels on the surface of cells (*n* = 3 patients) and **d** NExT derived from T-cell-enriched cultures re-activated and collected at 24 and 48 h (*n* = 3 patients) (green histograms) compared with their corresponding isotypes (pink histograms). **e** Mean of Fluorescence Intensity (MFI) fold change of surface PD1, LAG3, and TIM3 normalized with their corresponding (*n* = 3 patients). Data are represented as mean ± SEM. Comparison with baseline (0 h): *p < 0.05, **p < 0.01, and ****p < 0.0001

To ascertain the optimal time for isolating membranes from exhausted T-cells (ExT) for coating NPs, we conducted a study on the expression of PD1, LAG3, TIM3, and TIGIT markers associated with T-cell exhaustion in a model of acute activation in T-cell-enriched cultures from TNBC-patient-derived PBMCs (Fig. 2a). We found the higher expression levels at 24 (LAG3) and 48 h (TIGIT, TIM3, PD1), these being the time points selected to isolate the membranes of ExT for the further decoration of PLGA cores (Fig. 2b and Fig. S3). Given the expression levels observed in the previous experiment, PD1, LAG3, TIM3, and TIGIT were evaluated in different T-cell-enriched cultures from PBMCs of TNBC patients activated for 24 and 48 h. Our results indicated that PD1, LAG3, TIM3, and TIGIT were expressed in approximately 64%, 43%, 55%, and 6%, respectively, of the ExT analyzed (Fig. 2c and Fig. S4). We further confirmed the preservation, integrity, and proper orientation of PD1, LAG3, and TIM3 receptors after ExT membrane isolation and NP coating (NExT) from T-cell-enriched cultures of other TNBC patients. Similar to the profile obtained in ExT, we found that PD1 was the most prevalent receptor on the surface of NExT (∼ 50%), followed by TIM3 (∼ 30%) and LAG3 (∼ 27%) (Fig. 2d). TIGIT was barely detected on the surface of the NPs (∼ 2%) (Fig. S4). Further examination of normalized mean fluorescence intensity revealed consistent amounts of the IC receptors on NExT comparable to those of ExT (Fig. 2e).

### NExT achieve specific targeting to TNBC cells with PDL1 expression

**Fig. 3:**
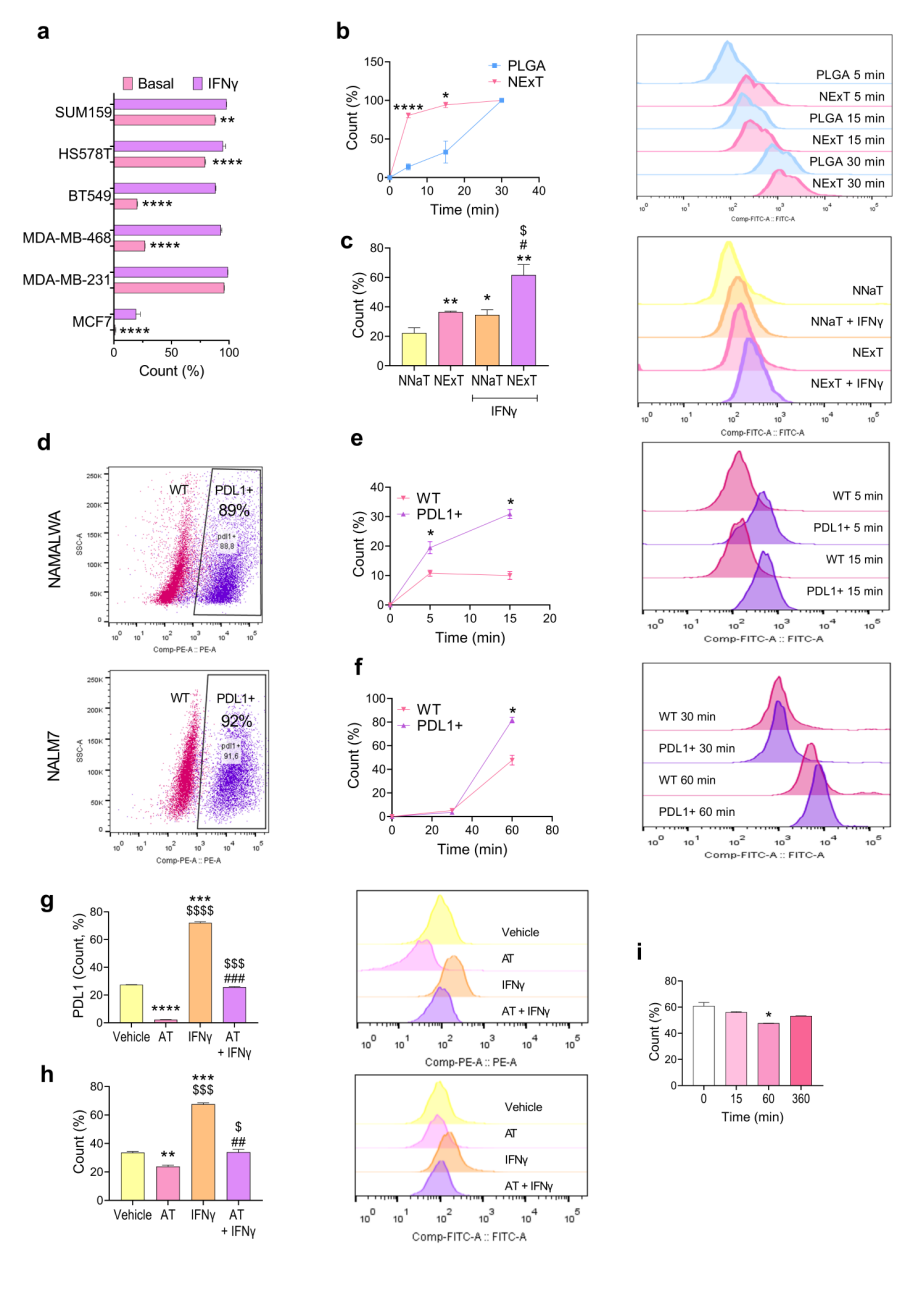
Functional characterization for tumor cell targeting by NExT. **a** Basal and IFNγ-induced (100 ng/ml for 24 h) PDL1 levels in different breast cancer cell lines. **b** Quantification and representative flow cytometry histograms of the percentage of cells positive for coumarin-6 after treatment with PLGA or NExT for 5 and 15 min in SUM159 cells (*n* = 2 patients). Comparison with PLGA: *p < 0.05 and ****p < 0.0001. **c** Quantification and representative flow cytometry histograms of the percentage of cells positive for coumarin-6 after treatment with PLGA NPs coated with membranes from T-cell enriched cultures (NNaT) or NExT in MDA-MB-468 cells stimulated or not with IFNγ (100 ng/ml for 24 h) for 15 min (*n* = 2 patients). Comparison with NNaT: *p < 0.05 and **p < 0.01; Comparison with NExT: $*p* < 0.05; Comparison with NNaT + IFNγ: #*p* < 0.05. **d** Representative dot plot of PDL1 levels in Namalwa and Nalm7 cells transduced with PDL1 plasmid (PDL1^+^) or wild type (WT). **e** Quantification and representative flow cytometry histograms of coumarin-6-positive cells after treatment with PLGA or NExT in Namalwa (WT and PDL1^+^) cells treated for 5 and 15 min, and **f** Nalm7 (WT and PDL1+) cells treated for 30 and 60 min (*n* = 2 patients). Comparison with WT: *p < 0.05. **g** Quantification and representative flow cytometry histograms of PDL1 levels and **h** percentage of coumarin-6-positive cells after treatment with NExT in MDA-MB-468 cells stimulated or not with IFNγ (100 ng/ml for 24 h) and blocked or not with atezolizumab (AT) (10 µg/ml for 24 h) for 15 min (*n* = 2 patients). Comparison with Vehicle: **p < 0.01, ***p < 0.001 and ****p < 0.0001; Comparison with AT: $$$*p* < 0.001 and $$$*p* < 0.0001; Comparison with IFNγ: ##*p* < 0.01 and ###*p* < 0.001. **i** PDL1 levels in SUM159 cells treated with NExT for 0, 15, 60, and 360 min. Comparison with basal levels (0 h): *p < 0.05. Data are represented as mean ± SEM.

Due to the consistent retention of PD1 on NExT (Fig. 2c-e), we hypothesized that PD1/PDL1 interaction could be a primary mechanism for the specific targeting and intratumor accumulation of NExT. To test this hypothesis, we initially examined the PDL1 levels in various breast cancer cell lines and found that all the TNBC cells tested showed higher basal and IFNγ-induced expression of PDL1 than MCF7 (ER^+^), as expected [27]. Among these, SUM159 and MDA-MB-231 cells exhibited the highest expression (Fig. 3a and Fig. S5). We then evaluated the capacity of NExT to target PDL1^high^ tumor cells by treating SUM159 cells with coumarin-6-loaded NExT and PLGA cores. As a result, we found faster targeting and higher numbers of coumarin-6-positive cells after treatment with NExT for 5 and 15 min (93.63% and 99.38%, respectively) compared with PLGA (33.42% and 43.46%, respectively). After 30 min, the maximum number of positive cells was reached in both groups (Fig. 3b). To further validate our hypothesis, we investigated the differential targeting capacity of NExT, in conditions of low and high PD1/PDL1 expression, in cells treated with coumarin-6-loaded PLGA NPs coated with membranes from T-cell enriched cultures (NNaT) or NExT. In MDA-MB-468 cells, exhibiting substantial responsiveness to IFNγ in terms of PDL1 expression (∼ 95%) compared to basal levels (∼ 20%) (Fig. 3a), NNaT (PD1^low^) treatment without stimulation with IFNγ (PDL1^low^) achieved the lowest number of positive cells (∼ 20%). Conversely, the treatment with NExT (PD1^high^) after stimulation with IFNγ (PDL1^high^) promoted a higher number of targeted cells (∼ 40%), which was similar to the treatment with NNaT and stimulation with IFNγ (PD1^low^/PDL1^high^). Notably, the highest number of targeted cells (∼ 70%) resulted from the treatment with NExT and the subsequent activation with IFNγ (PD1^high^/PDL1^high^) (Fig. 3c). Flow cytometry analysis in genetically engineered Namalwa and Nalm7 cell lines transduced with PDL1 and treated with coumarin-6-loaded NExT confirmed the avidity of NExT for PDL1^high^ tumor cells (Fig. 3d). Both cell lines exhibited a greater number of targeted cells in the PDL1^+^ population compared to wild type (WT) (Fig. 3e, f). We validated our hypothesis through a rescue experiment in MDA-MB-468 cells treated with the anti-PDL1 antibody atezolizumab, which is approved by the FDA for the treatment of TNBC [43], and coumarin-6-loaded NExT upon stimulation with IFNγ. As expected, atezolizumab efficiently blocked both basal and IFNγ-induced PDL1 levels (Fig. 3g), which abrogated the tumor cell targeting by NExT. As a result, the number of coumarin-6-positive cells targeted by NExT after stimulation with IFNγ (∼ 70%) was decreased up to basal levels (∼ 35%) after the treatment with atezolizumab (Fig. 3h). Moreover, our findings revealed that SUM159 cells treated with NExT displayed lower PDL1 expression compared to the control (Fig. 3i). Overall, these results support that PD1/PDL1 interaction could be the primary mechanism of NExT to target tumor cells.

### Enhanced in vitro therapeutic efficacy of NExT

**Fig. 4:**
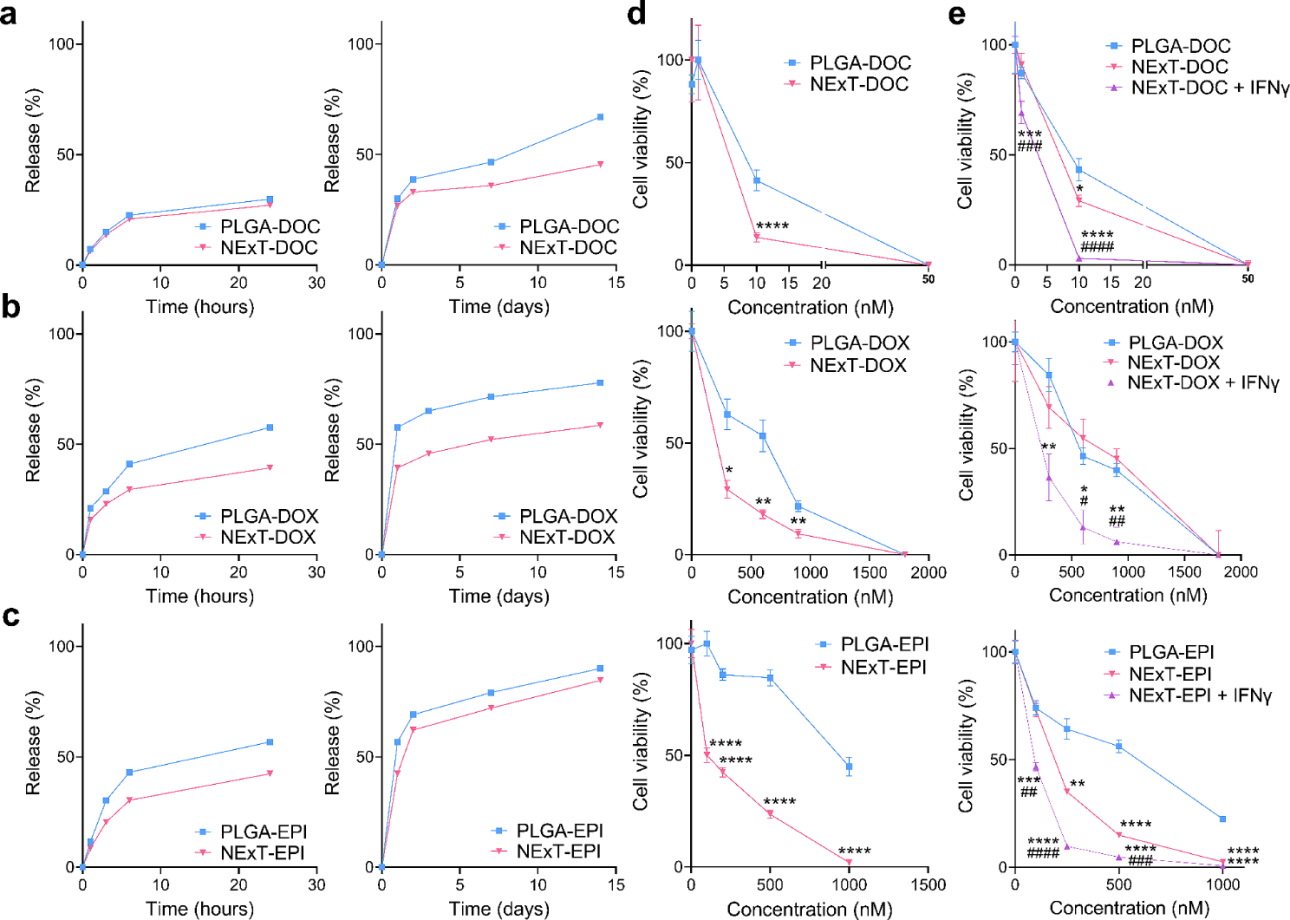
In vitro therapeutic efficacy of NExT. **a**In vitro release of docetaxel (DOC), **b** doxorubicin (DOX), and **c** epirubicin (EPI) for 1, 3, 6, and 24 h (left), and 1, 3, 7, and 14 days (right) in PBS Tween (0.1%) (pH 7.4) at 37 °C. **d** Cell proliferation of SUM159 cells and **e** MDA-MB-468 cells, stimulated or not with IFNγ (100 ng/ml), treated with PLGA NPs and NExT loaded with DOC, DOX, and EPI for 48 h (*n* = 3 patients, four replicates). Data are represented as mean ± SEM. Comparison with PLGA: *p < 0.05, **p < 0.01, ***p < 0.001, and ****p < 0.0001; Comparison with NExT: #*p* < 0.05, ##*p* < 0.01, ###*p* < 0.001, and ####*p* < 0.0001

To assess the efficacy of cancer-cell-targeted drug delivery of chemotherapy-loaded NExT, we formulated PLGA NPs loaded with DOC, DOX, and EPI. The physicochemical characterization, EE, and LC are shown in Table S1. We further prepared NExT from the chemotherapy-loaded PLGA cores and investigated their cumulative release kinetics over 14 days. Our findings revealed a drug release pattern resembling the typical biphasic profile observed in PLGA NPs, albeit with a more gradual release in NExT (Fig. 4a-c). The assessment of the antiproliferative effects of DOC, DOX, and EPI loaded in PLGA cores and NExT was conducted in SUM159 and MDA-MB-468 cells. Treatment with NExT loaded with DOC, DOX, and EPI notably reduced the viability of SUM159 cells compared to these drugs encapsulated in PLGA (Fig. 4d). Similarly, the cytotoxicity of DOC and EPI in MDA-MB-468 cells exhibited an increase when delivered via NExT, while no discernible difference was noted for DOX. However, their therapeutic efficacy was augmented upon IFNγ stimulation (Fig. 4e).

### NExT is a non-toxic platform with remarkable intratumor accumulation

**Fig. 5:**
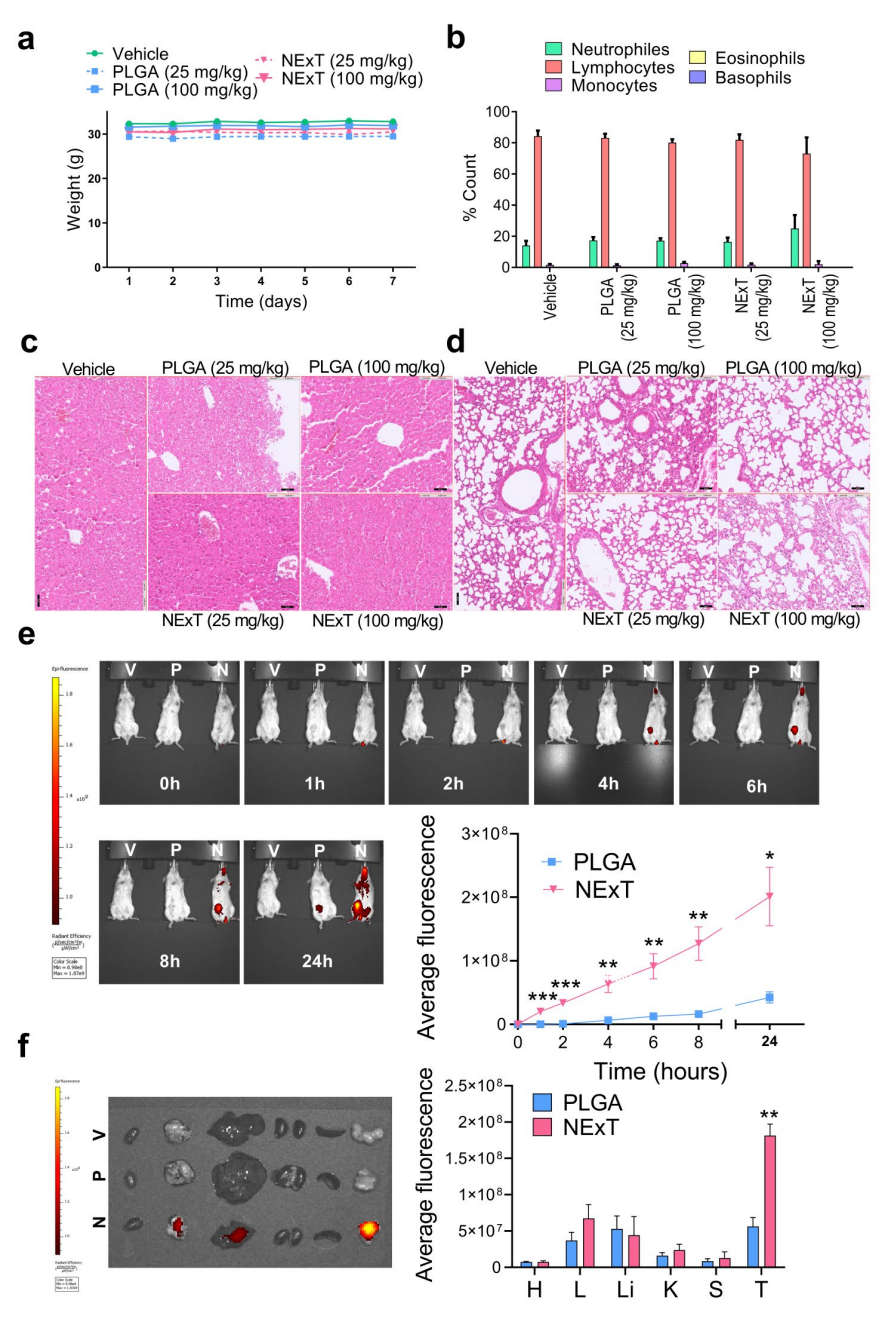
In vivo safety, distribution and tumor targeting of NExT. **a** Weight, **b** Leukocyte formula, and **c** Representative images of livers and **d** lungs stained with hematoxylin/eosin (H&E) of CD1 mice treated with Vehicle, empty PLGA (25 and 100 mg/kg), and empty NExT (25 and 100 mg/kg) (*n* = 3 mice/group). Scale bar = 50 μm. **e** Representative images and in vivo quantification of intratumor accumulation of fluorescence in SUM159-xenograft mice treated with IR780-loaded PLGA (P) or NExT (N) or Vehicle (V) for 0, 1, 2, 4, 6, 8, and 24 h (*n* = 6 mice/group). **f** Representative images and quantification of fluorescence in the organs (Heart: H; Lung: L; Liver: Li; Kidneys: K; Spleen: S) and tumor (T) of mice treated with IR780-loaded PLGA (P) or NExT (N) or Vehicle (V) for 24 h (*n* = 6 mice/group). Data are represented as mean ± SEM. *p < 0.05, **p < 0.01, and ***p < 0.001

The autologous patient-derived NExT platform is envisioned as a vehicle for adoptive nanotherapy that must exhibit good biosafety profiles for future applications in humans. In pursuit of this objective, in vivo toxicity was assessed through histopathological and hematological analysis in CD1 mice injected intravenously with 25 mg/kg or 100 mg/kg of NExT and PLGA. The animals treated with NPs did not show significant changes in body weight (Fig. 5a), leukocyte formula (Fig. 5b), or other hematological parameters (Table S2). No obvious pathological alterations were found in organs like the liver (Fig. 5c) or lungs (Fig. 5d) of mice treated with NPs compared to the vehicle group.

The tumor-targeting efficacy of NExT was examined by the in vivo biodistribution of IR780-loaded NExT and PLGA injected intravenously in a SUM159 xenograft mouse model. In vivo fluorescence imaging revealed faster and more selective accumulation and penetration in the tumor site of NExT than PLGA. Notably, fluorescence of the NExT group was detectable as early as 1 h post-injection with no decrease during the test time. By 24 h, the fluorescence was more than 5-fold higher than that of PLGA. In contrast, PLGA exhibited slower and lower intratumor distribution, with fluorescence only becoming detectable from 4 h (Fig. 5e). Examination of tissues ex vivo at 24 h displayed a similar biodistribution pattern between NExT and PLGA in major organs, which were predominantly found in the liver. Importantly, it was confirmed that NExT were primarily localized in the tumor (Fig. 5f).

### In vivo therapeutic activity of NExT in a PDL1^+^ PDX model of TNBC

**Fig. 6:**
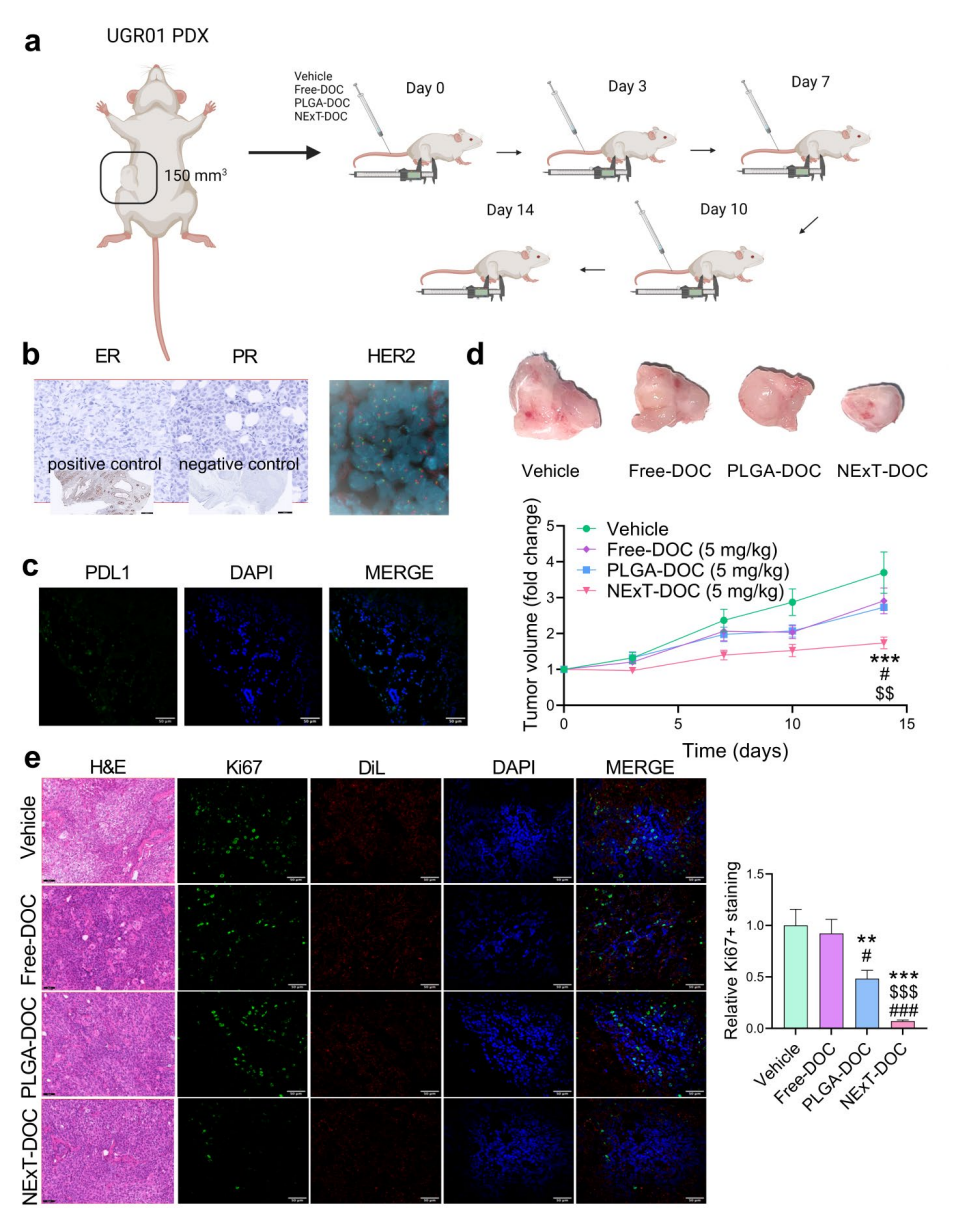
Therapeutic efficiency of NExT in a PDX model of TNBC. **a** Schematic illustration of the therapeutic schedule for the PDX mouse model (UGR01). **b** Immunohistochemistry of ER (estrogen receptor) and PR (progesterone receptor) and assessment of negative HER2 amplification by FISH showing two copies of the gene (red) and centromere 17 (green) per nucleus (blue). Scale bar = 500 μm. **c** Representative confocal images of PDL1 (green) in the PDX model (UGR01) (original optical objective: 40×). Scale bar = 50 μm. **d** Tumor volume fold change of UGR01-PDX-bearing mice treated with Vehicle, free docetaxel (Free-DOC), docetaxel-loaded PLGA (PLGA-DOC), and docetaxel-loaded NExT (NExT-DOC) (*n* = 5 mice/group) and representative images of excised tumors at day 14. **e** Representative confocal images (original optical objective: 40×), H&E, and quantification (*n* = 5 mice/group) of Ki67 (green) in UGR01 PDX tumors. DiL was used to stain cell membranes. Scale bar = 50 μm. Data are represented as mean ± SEM. Comparison with Vehicle: **p < 0.01, and ***p < 0.001; Comparison with Free-DOC: #*p* < 0.05, and ###*p* < 0.001; Comparison with PLGA-DOC: $$*p* < 0.01, and $$$*p* < 0.001

As NExT demonstrated exceptional intratumor accumulation and remarkable therapeutic efficacy in PDL1^+^ TNBC cells, we conducted further investigations into the therapeutic potential of autologous patient-derived NExT in a PDL1^+^ tumor-in-mouse model derived from a TNBC patient (UGR01). PDX-bearing mice (*n* = 5 per group) were injected with docetaxel, free or encapsulated in PLGA and NExT (coated with T-cells from the same source patient of the PDX) at a dose of 5 mg/kg at days 0, 3, 7, and 10 for a total dose of 20 mg/kg. The changes in tumor size were monitored up to day 14 (Fig. 6a). Immunohistochemistry and FISH validated the UGR01 PDX model to be TNBC, mirroring the characteristics of the source patient (Fig. 6b). In addition, confocal microscopy indicated that the PDX tissue was PDL1^+^ (Fig. 6c), a status that can predict the benefit of being treated with NExT-encapsulated therapy. After 14 days, the mice treated with DOC-loaded NExT exhibited significantly diminished tumor growth compared to those receiving the free drug, PLGA-DOC and Vehicle. Expectedly, the PLGA-DOC and Free-DOC groups did not demonstrate significant therapeutic activity compared to Vehicle (Fig. 6d), as seen before with a cumulative dose of 25 mg/kg [44]. Confocal microscopy data indicated a substantial reduction of Ki67 by NExT-DOC compared to the other groups, suggesting that NExT improved the inhibition of tumor proliferation by DOC (Fig. 6e).

**Fig. 7:**
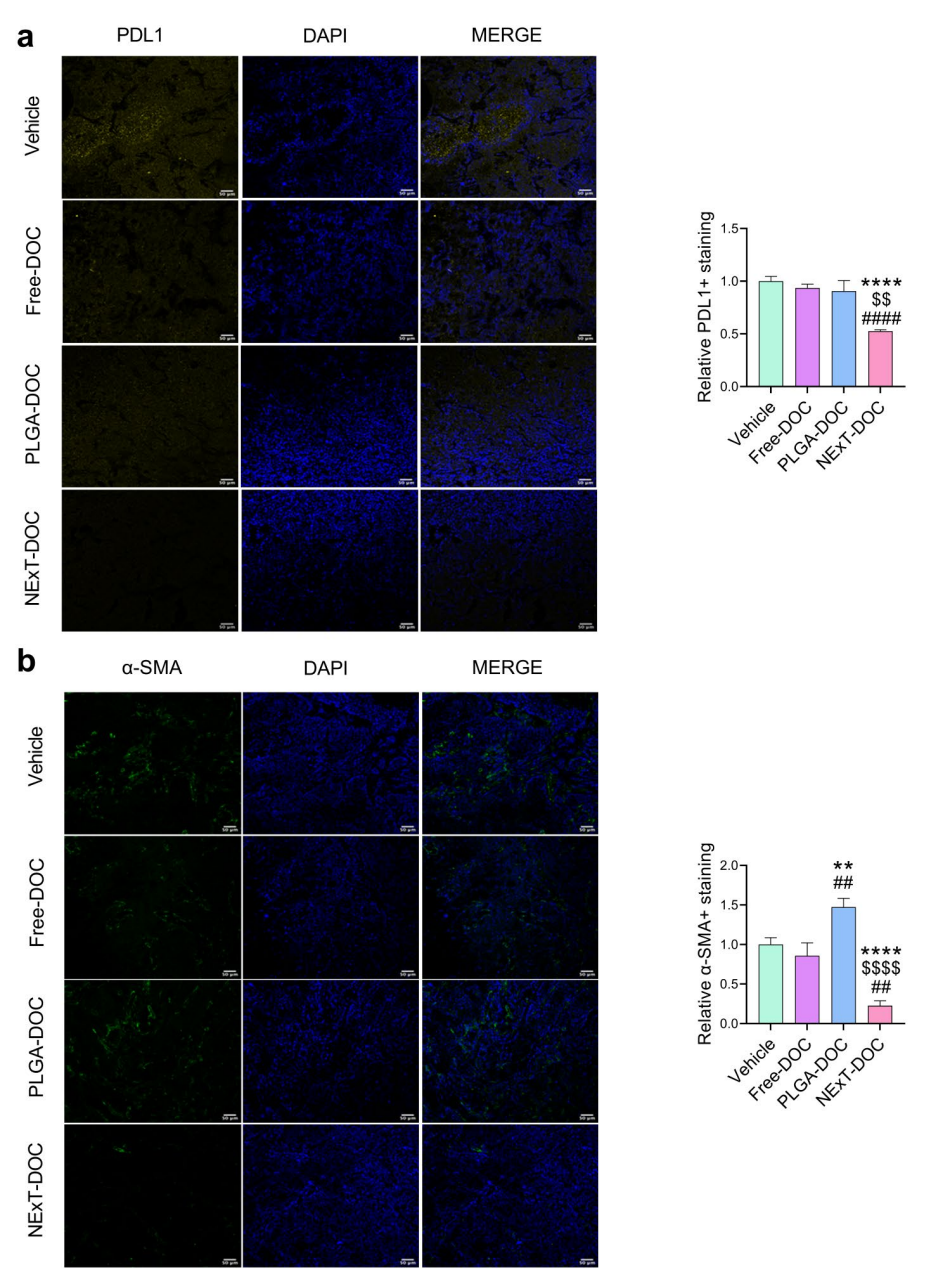
PDL1 and stromal α-SMA expression in the PDX tumor tissue. **a** Representative confocal images (original optical objective: 20×) and quantification of PDL1 and **b** stromal α-SMA in UGR01 PDX tumors (*n* = 3 mice/group). Scale bar = 50 μm. Comparison with Vehicle: **p < 0.01, and ****p < 0.0001; Comparison with Free-DOC: ##*p* < 0.01, and ####*p* < 0.0001; Comparison with PLGA-DOC: $$*p* < 0.01, and $$$$*p* < 0.0001

Because our in vitro experiments indicated that NExT can target tumor cells through the disruption of the PD1/PDL1 axis (Fig. 3g-i), we further confirmed the PDL1 levels in tumor cells and the TME in the PDX tumor tissue. Confocal microscopy showed a significant decrease in the expression of PDL1 (both in tumor cells and TME) by NExT-DOC compared to PLGA-DOC and Free-DOC groups, which did not differ from Vehicle (Fig. 7a). These results were correlated with a significant reduction in cancer-associated fibroblasts (CAFs) as evidenced by the marker α-smooth muscle actin (α-SMA) (Fig. 7b).

## Discussion

PLGA NPs are frequently chosen as cores of BNPs due to their favorable performance when coated with cell membranes [4, 6, 7, 10, 12, 13, 18, 19]. Our results showed that PLGA cores were between 100 and 300 nm in size, which is optimal for drug encapsulation to ensure the balance between an adequate drug load and the promotion of enhanced permeability and retention effect, therefore maximizing the desired cytotoxic effects [26, 30]. After confirming that our culture protocol generates adequate numbers of T-cells for coating from PBMCs of TNBC patients, as expected [45], NExT NPs were prepared. Physicochemical analysis revealed that NExT were larger (ranging from 5 to 22 nm), as expected [4, 7–13], with the typical core-shell structure, and a ζ-potential around − 20 mV as reported by other studies in membrane-coated NPs, which was correlated with good stability, high cytotoxic effect, and reduced opsonization [26, 46]. Additionally, consistent with previous reports [47], we confirmed the stability for prolonged periods and the good biocompatibility of the coated NPs. Collectively, our results confirm the successful decoration of PLGA NPs, and validate our protocol for membrane extraction and purification, aligning consistently with other similar methodologies [12, 13]. Additionally, these findings endorse the suitability of our culture protocol in generating large-scale numbers of patient-derived T-cell-enriched cultures.

Tumor growth or chronic viral infections cause a persistent immune activation resulting in T-cell dysfunction or exhaustion. This state is characterized by heightened expression of inhibitory checkpoint receptors such as PD1, LAG3, and TIM3, alongside diminished cytokine production and proliferation. In vitro models of T-cell exhaustion by chronic activation are complex and yield significantly lower cell numbers than acute activation. However, the high expression of inhibitory checkpoint receptors can be transiently achieved after T-cell activation without becoming dysfunctional and maintaining the cell number [48, 49]. Our results underscore the good performance of our methodologies to produce modified cells (ExT) without requiring advanced engineering techniques. Positioned as the cornerstone of adoptive nanotherapy, patient-derived NExT are intended to deliver therapies in a customized way for each patient after their autologous infusion to the same individual. Consequently, genetic variations contributing to heterogeneous expression of IC receptors among patients [50] are expected to introduce variability in receptor expression profiles on T-cells, ExT, and therefore NExT, as evidenced in our study (Fig. 2c-e). Active targeting is significantly improved by NPs that display multiple specific molecules on their surface, mainly because they can bind to different target molecules simultaneously, thereby increasing their avidity for the target cells [51]. Accordingly, we demonstrated herein that NExT are naturally functionalized with diverse receptors (PD1, LAG3, TIM3) that can interact with their corresponding ligands (e.g., PDL1, Galectin-3, FGL-1, MHCII, Galectin-9, HMGB1, Ceacam-1) within the tumor. Moreover, while not within the scope of this study, we cannot disregard the potential decoration of NExT with TCR capable of recognizing MHCI/II expressed on tumor cells [6, 7]. Overall, this enables a higher and more specific intratumor accumulation of NPs and therefore improved drug delivery and efficacy.

TNBC tumors express specific ligands of PD1, LAG3, and TIM3 such as PDL1, Galectin-3, FGL-1, MHCII, Galectin-9, or secreted HMGB1 [52–55], which offer multiple targets for the improved active drug delivery by NExT. While the presence of other molecules like LAG3 or TIM3 on NExT can improve tumor targeting and accumulation, our focus remained on PD1 due to its consistent retention on NExT (Fig. 2c-e), identifying PD1/PDL1 interaction as a primary mechanism for specific targeting and intratumor accumulation, and supporting the relevance of PD1/PDL1 interaction for NExT specificity in targeting PDL1^+^ tumors (Fig. 3), as observed in prior studies with other platforms [56, 57]. Moreover, the lower PDL1 expression in SUM159 cells treated with NExT (Fig. 3i) not only indicated interference of the nanoplatform with anti-PDL1 antibody binding to PDL1 on tumor cells but also suggested the disruption of the PD1/PDL1 axis. Analogously, earlier studies have highlighted the increased affinity of BNPs for target cells via other or unknown interactions [5, 6, 9–11, 13, 14, 58–60]. Although other reports have explored the effectiveness of nanoplatforms conjugated with antibodies or engineered vesicles to disrupt the PD1/PDL1 inhibitory axis [2, 21, 56, 57, 61–63], studies using BNPs to selectively target cancer cells through immune exhaustion [16, 17], and exploiting tumor IE, are lacking. This situation positions NExT as a novel class of BNPs for active drug delivery, boasting characteristics akin to IT.

Currently, chemotherapy using agents like taxanes or anthracyclines remains the established approach for unresectable TNBC. However, these therapies pose challenges due to their non-selective targeting of tumor tissue, resulting in adverse events, low biodistribution rates, and efficacy issues [28, 51]. We assessed the efficacy of NExT loaded with DOC, DOX, and EPI in vitro. First, consistent with earlier studies, we found a biphasic drug release pattern resembling the typical biphasic profile observed in PLGA NPs, this being more gradual in NExT due to the membrane acting as a barrier that impedes drug diffusion [4, 13, 64]. Further, compared with PLGA NPs, our results underscore the superior precision and efficacy in delivering and boosting the therapeutic index of different chemotherapeutic drugs by NExT, as well as their higher therapeutic efficiency in PDL1^+^ TNBC cells, likely due to heightened avidity of NExT resulting from the increased interaction with more surface-bound PDL1 molecules, as observed in reports on NPs conjugated with PDL1 antibodies or peptides [56, 57]. Certain chemotherapies, such as those examined here, potentiate antitumor immunity through mechanisms like the induction of immunogenic cell death or the upregulation of IC receptors and ligands, including PDL1, thereby boosting the IC-based IT, which is the core of chemoimmunotherapy [65, 66]. Consequently, it is plausible that the targeted specificity of NExT to PDL1^low^ cells can be amplified by the chemotherapy entrapped inside by upregulating IC ligands (e.g., PDL1) and synergizing with IT as reported to other NPs [67].

As previously reported for other BNPs [7, 11, 14, 16, 47], our data demonstrates the good biocompatibility of NExT. In terms of biodistribution in vivo, among major organs, NExT and PLGA were mainly found in the liver as reported for other PLGA BNPs [6, 7, 19] and PDL1/PD1-targeting nanosystems [2, 21, 57, 61–63]. Importantly, we found that NExT were fundamentally accumulated within tumor tissue. Overall, these results strongly indicate the superior and more precise tumor-targeting capability of NExT compared to PLGA, which endorses their future application in clinics for cancer therapy. The in vivo therapeutic efficacy of NExT was determined in a PDL1^+^ PDX model of TNBC. As expected [18], our findings highlight that NExT serves as a nanoplatform that efficiently targets PDL1^+^ tumors. Notably, the cumulative dose of DOC injected in this study was three times lower than that administered to PDX models of TNBC or encapsulated in PLGA NPs [37, 40], indicating that NExT significantly boost the therapeutic index of chemotherapy.

Chemoimmunotherapy, known as the combination of chemotherapy and IC inhibitors such as atezolizumab (anti-PDL1) or pembrolizumab (anti-PD1), is an advance in first-line cancer therapy, including for TNBC patients [43, 68]. Our results indicated that NExT bind to PDL1 on tumor cells and reduce their availability (Fig. 3i), suggesting that NExT could elicit a PDL1 occupancy that would block PD1/PDL1 interaction between T-lymphocytes and tumors. Indeed, we found reduced PDL1 levels in cancer cells and TME of the PDX tumor tissue treated with NExT-DOC (Fig. 7a). It is known that some BNPs can modulate the TME to enhance the antitumor immunity and reduce the immunosuppressive microenvironment by the activation of immune cells through the blockade of PD-1/PD-L1 interaction [17, 20, 69, 70]. Disruption of the PD-1/PD-L1 axis has been reported as the mechanism to alter the TME by other PD1-expressing platforms and immune-checkpoint-based IT [21, 61, 71], suggesting that the interference of NExT with PDL1 could remodel the TME. In this sense, as part of the TME, the tumor stroma induces cancer progression, metastasis, and resistance to therapy. Within the tumor stroma, CAFs are one of the most abundant cell populations that promote tumorigenesis, metastasis, recurrence, drug resistance, immunosuppressive TME by expressing PDL1, and poor patient prognosis in several cancers [72, 73]. α-SMA is a specific marker of CAFs that is correlated with metastatic disease and poor prognosis in TNBC patients [74]. Similar to other nanoplatforms conjugated with docetaxel [75], our investigations showed that NExT significantly reduced stromal α-SMA in the PDX tumor tissue (Fig. 7b), suggesting that NExT not only can target tumor cells but also remodel the TME by the depletion of CAFs through the interaction with PDL1. Hence, future investigations are warranted to ascertain whether NExT might function as an autologous IT by disrupting the PD1/PDL1 axis, augmenting the anticancer immune response without immunogenicity, similar to findings reported for other platforms with immunotherapeutic properties [2, 21, 56, 57, 61–63]. If confirmed, we can hypothesize that chemotherapy-loaded NExT might elicit effects comparable to those of chemoimmunotherapy as reported [62], which enhances the interest in the clinical application of this nanoplatform. On this basis, we reasonably question whether early-stage TNBC patients would benefit from NExT regardless of PDL1 status as reported for atezolizumab [76], or it would be of interest for the treatment of advanced disease with PDL1 positivity, where a careful choice of chemotherapeutics should be made first to facilitate the upregulation of PDL1 in the tumor tissue to increase the tumor sensitivity to NExT [65].

## Conclusion

**Fig. 8:**
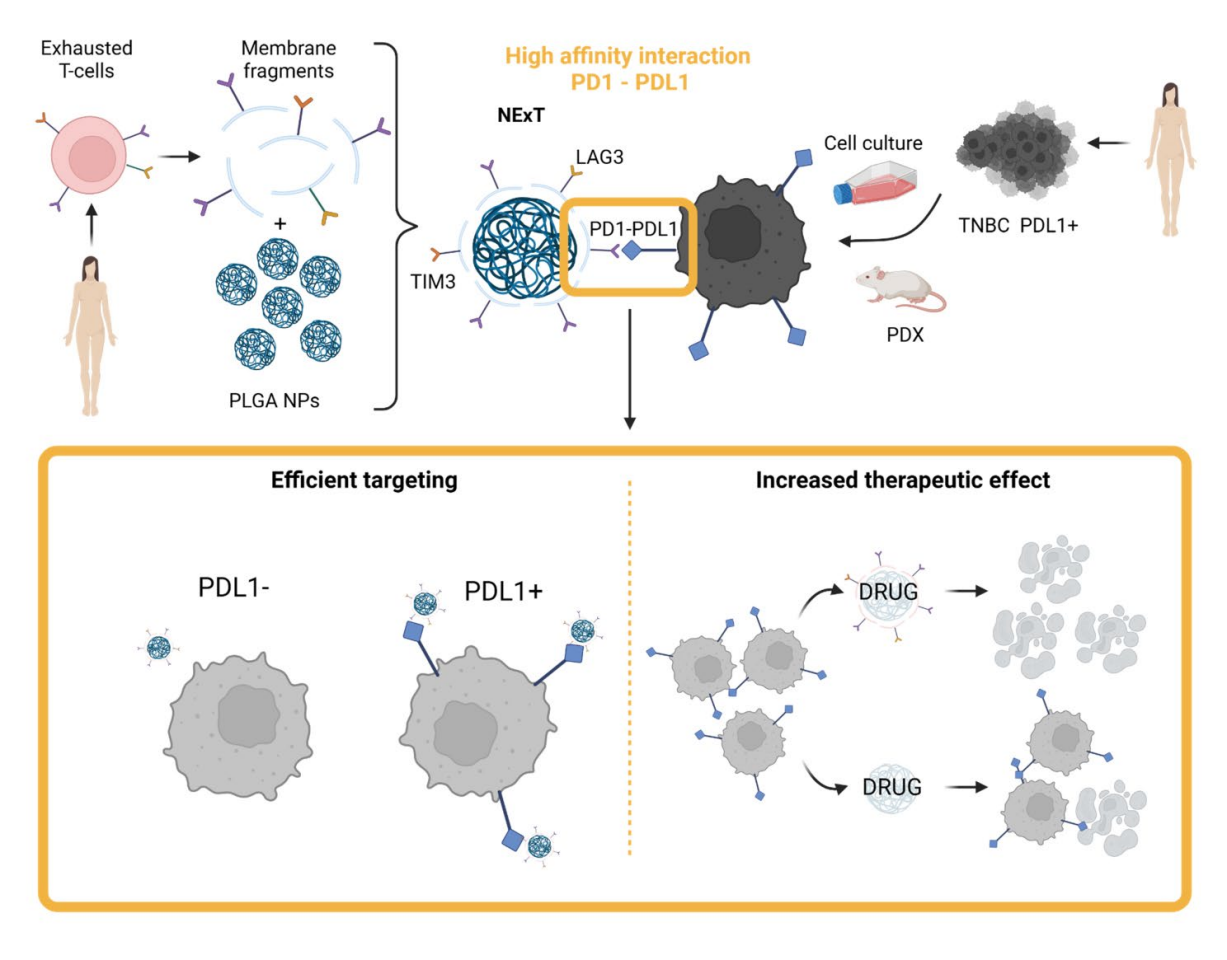
Schematic illustration of conclusions

In the present study, we present a pioneering active drug delivery approach that converts IE, as a strength of cancer cells, into a vulnerability to achieve an effective targeted therapy. We have successfully developed NExT, a novel class of BNPs coated with membranes of TNBC-patient-derived exhausted T-lymphocytes, that simulate the interplay between cancer cells and the immune system, leading IE, to specifically target tumors. Through the transient activation of T-cell-enriched cultures from TNBC-derived PBMCs, we have established that the NExT platform is naturally functionalized with the IC receptors PD1, TIM3, and LAG3 that can bind the cognate ligands on tumor cells. Patient-derived NExT exhibited high specificity for PDL1^+^ tumor cells, primarily attributed to the PD1/PDL1 interaction, which boosts intratumor accumulation, selective active targeting, and the therapeutic index and efficiency of chemotherapeutic drugs, as we showed in a PDL1^+^ PDX model of TNBC. These advantages, coupled with their demonstrated good biocompatibility in vivo, the lack of complex and sophisticated engineering methods used herein, and their potential as autologous disruptors resembling IT by targeting the PD1/PDL1 axis that can remodel the TME, uphold the wider future clinical application of patient-derived NExT for the autologous treatment, including chemoimmunotherapy, of cancer patients with PDL1^+^ tumors through a personalized adoptive nanotherapy (Fig. 8).

### Electronic supplementary material

Below is the link to the electronic supplementary material.

Supplementary Material 1

Supplementary Material 2

Supplementary Material 3

## Data Availability

The data that support the findings of this study are available from the corresponding author upon reasonable request.

## Abbreviations

BNPs: Biomimetic NPs
DCM: Dichloromethane
DLS: Dynamic Light Scattering
DOC: Docetaxel
DOX: Doxorubicin
EE: Encapsulation Efficiency
EPI: Epirubicin
ExT: Exhausted T-lymphocytes
IC: Immune Checkpoint
IE: Immune Evasion
IT: Immunotherapy
LC: Loading Capacity
NaT: T-cell-enriched cultures
NExT: Nanoparticles of Exhausted T-cells
NNaT: NPs coated with membranes from NaT
NPs: Nanoparticles
PDX: Patient-Derived Xenograft
PVA: Polyvinyl Alcohol
TEM: Transmission Electron Microscopy
TME: Tumor Microenvironment
TNBC: Triple-Negative Breast Cancer
