# Supplementary material for: Autologous patient-derived exhausted nano T-cells exploit tumor immune evasion to engage an effective cancer therapy
Source: Mol Cancer. 2024 May 9;23:83. doi: 10.1186/s12943-024-01997-x (PMC11084007; doi:10.1186/s12943-024-01997-x)
Supplement: Supplementary file 1 — Supplementary Material 1 [file 12943_2024_1997_MOESM1_ESM.pdf]

Additional file 1

Supplementary Figures and Legends

Autologous patient-derived exhausted nano T-cells exploit tumor immune evasion to engage an effective cancer therapy.

José L. Blaya-Cánovas[1,2,3,#], Carmen Griñán-Lisón[2,3,4,5,#], Isabel Blancas[2,6,7], Juan A. Marchal[2,5,8,9], Cesar Ramirez-Tortosa[2,10], Araceli López-Tejada[2,3,4], Karim Benabdellah[3], Marina Cortijo-Gutiérrez[3], M.Victoria Cano-Cortés[2,3,11], Pablo Graván[2,5,12], Saúl A. Navarro-Marchal[2,5,8,12], Jaime Gómez-Morales[13], Violeta Delgado-Almenta[3], Jesús Calahorra[1,2,3], María Agudo-Lera[3], Amaia Sagarzazu[3], Carlos J. Rodríguez-González[6], Tania Gallart-Aragón[7,14], Christina Eich[15], Rosario M. Sánchez-Martin[2,3,11], Sergio Granados-Principal[2,3,4,*].

[1]UGC de Oncología Médica, Hospital Universitario de Jaén, 23007, Jaén, Spain

[2]Instituto de Investigación Biosanitaria ibs.GRANADA, University Hospitals of Granada-University of Granada, 18100, Granada, Spain

[3]GENYO, Centre for Genomics and Oncological Research, Pfizer/University of Granada/Andalusian Regional Government, 18016, Granada, Spain

[4]Department of Biochemistry and Molecular Biology 2, Faculty of Pharmacy, University of Granada, Campus de Cartuja s/n, 18071, Granada, Spain

[5]Excellence Research Unit "Modeling Nature" (MNat), University of Granada, 18100, Granada, Spain.

[6]UGC de Oncología, Hospital Universitario "San Cecilio", 18016, Granada, Spain

[7]Department of Medicine, University of Granada, 18016, Granada, Spain

[8]Biopathology and Regenerative Medicine Institute (IBIMER), Centre for Biomedical Research, (CIBM) University of Granada, 18100, Granada, Spain.

[9]Department of Human Anatomy and Embryology, Faculty of Medicine, University of Granada, 18016, Granada, Spain.

[10]UGC de Anatomía Patológica Hospital San Cecilio de Granada, 18016, Granada, Spain

[11]Department of Medicinal & Organic Chemistry and Excellence Research Unit of "Chemistry Applied to Biomedicine and the Environment", Faculty of Pharmacy, University of Granada, Campus de Cartuja s/n, 18071, Granada, Spain

[12]Department of Applied Physics, Faculty of Science, University of Granada, 18071, Granada, Spain

[13]Laboratorio de Estudios Cristalográficos IACT-CSIC-UGR, 18100, Armilla, Spain

[14]UGC de Cirugía General y del Aparato Digestivo, Hospital Universitario "San Cecilio", 18016, Granada, Spain

[15]Translational Nanobiomaterials and Imaging, Department of Radiology, Leiden University Medical Center, 2333, Leiden, the Netherlands

#These authors contributed equally

*Corresponding author: sergiogp@ugr.es

Supplementary Fig. S1

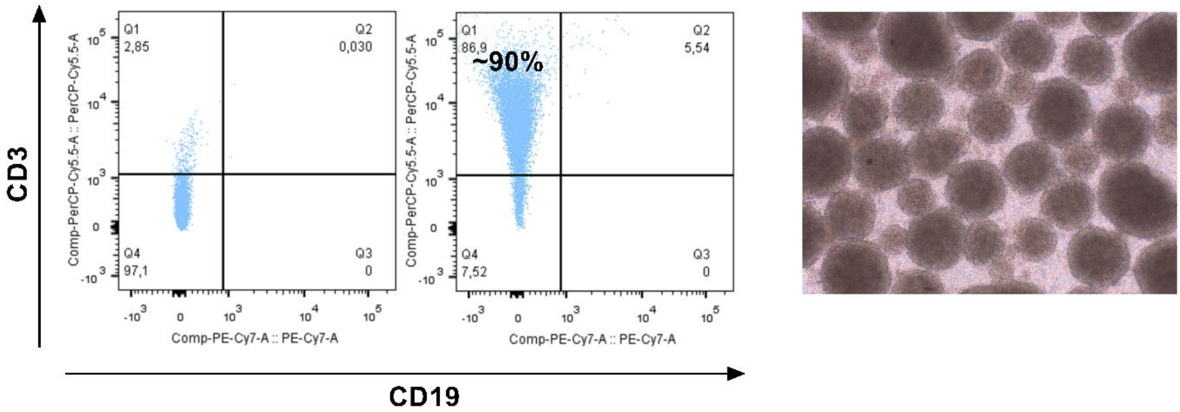

Figure S1. PBMC-derived T-cell-enriched cultures from TNBC patients. Representative flow cytometry dot plot of T-cell-enriched cultures of PBMCs, derived from TNBC patients after 3 weeks of expansion, stained with anti-CD3 and anti-CD19, and a representative image of a T-cell culture.

Supplementary Fig. S2

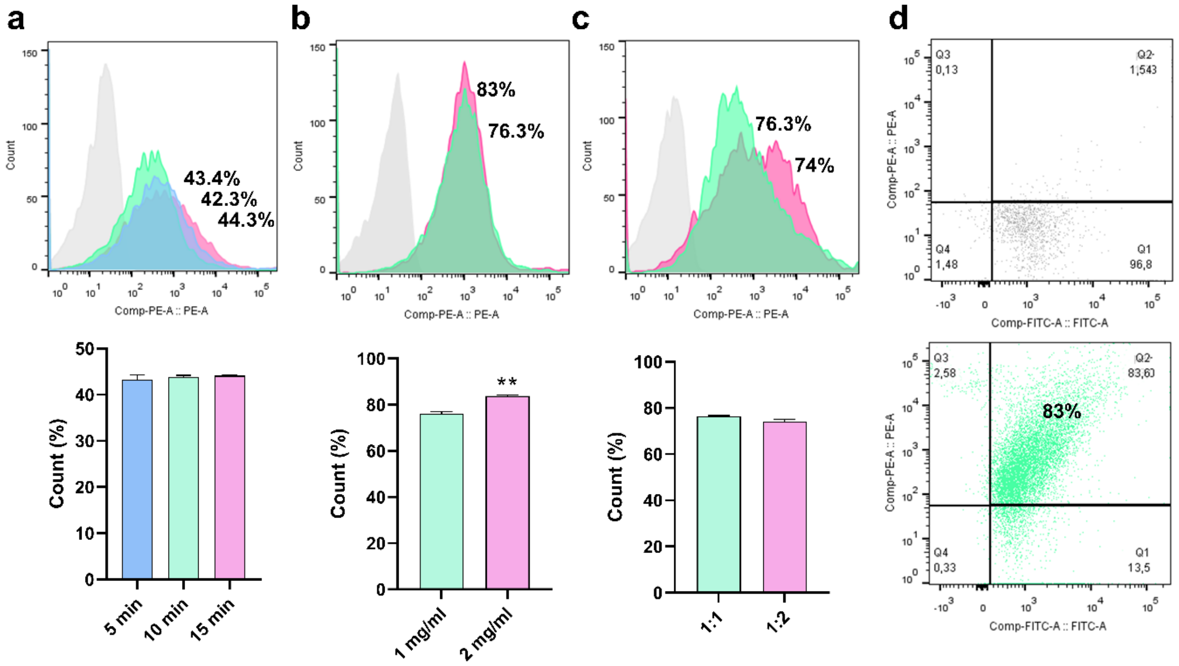

Figure S2. Optimization of the coating process. a Representative flow cytometry histogram and quantification of NExT coated with DiL-stained membranes after 5, 10, or 15 min of sonication (n=2 patients). **b** Representative flow cytometry histogram and quantification of NExT coated with DiL-stained membranes at different protein concentrations: 1 or 2 mg/ml and quantification (n=2 patients). **c** Representative flow cytometry histogram and quantification of NExT coated with DiL-stained membranes 1:1 or 1:2 PLGA:membrane volume ratios (n=2 patients). **d** Representative flow cytometry dot plot of PLGA FITC+ NPs (upper plot) and NExT FITC+ coated with DiL-stained membranes (lower plot). Data are represented as mean ± SEM. **p*<0.05, ***p*<0.01, and ****p*<0.001.

Supplementary Fig. S3

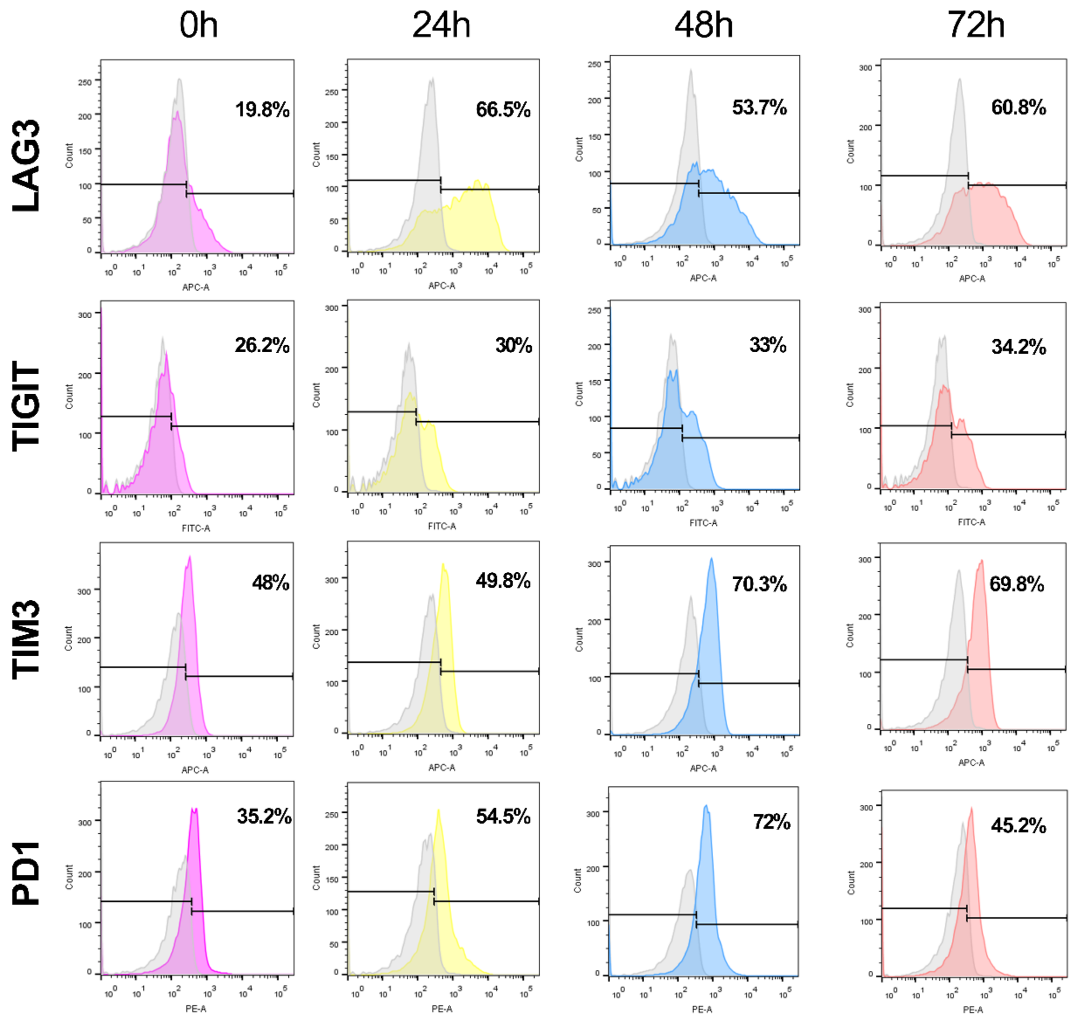

Figure S3. Characterization of T-cell exhaustion. Representative flow cytometry histograms of LAG3, TIGIT, TIM3, and PD1 levels, and isotype (grey), in T-cell-enriched cultures after activation with TransAct at 0, 24, 48, or 72h (n=4 patients).

Supplementary Fig. S4

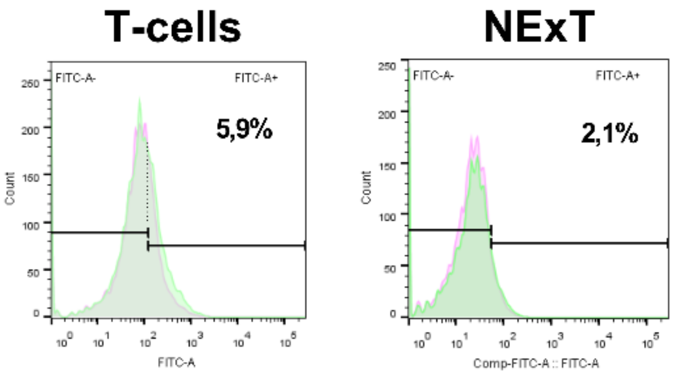

Figure S4. TIGIT expression on T-cells and NExT. Representative flow cytometry histograms of TIGIT on the surface of patient-derived T-cell-enriched cultures (n=3 patients) and NExT derived from T-cell-enriched cultures re-activated and collected at 24 and 48h (n=3 patients).

Supplementary Fig. S5

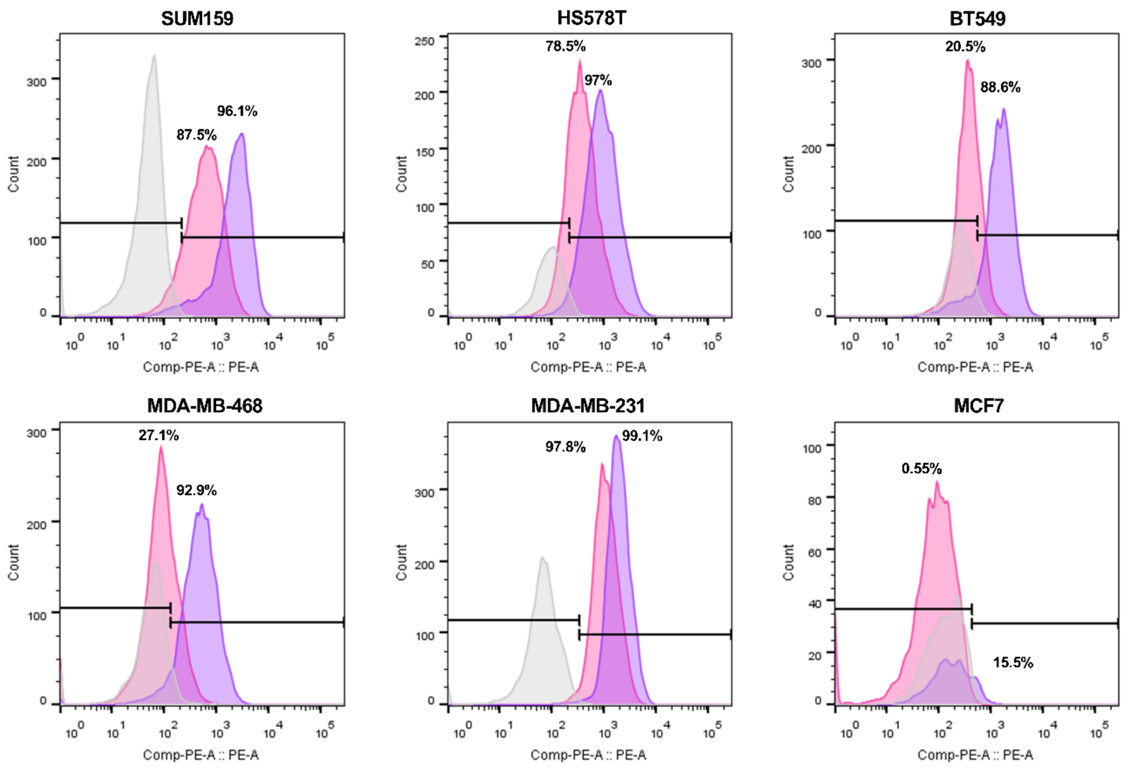

Figure S5. PDL1 levels in breast cancer cell lines. Representative flow cytometry histograms of basal (pink), IFNγ-induced (100 ng/ml for 24h) (purple) PDL1 levels, and isotype (grey) in SUM159, HS578T, BT549, MDA-MB-468, MDA-MB-231, and MCF7 cells.
