# Supplementary material for: Autologous patient-derived exhausted nano T-cells exploit tumor immune evasion to engage an effective cancer therapy
Source: Mol Cancer. 2024 May 9;23:83. doi: 10.1186/s12943-024-01997-x (PMC11084007; doi:10.1186/s12943-024-01997-x)
Supplement: Supplementary file 2 — Supplementary Material 2 [file 12943_2024_1997_MOESM2_ESM.pdf]

Additional file 2
Supplementary Tables

Autologous patient-derived exhausted nano T-cells exploit tumor immune evasion to engage an effective cancer therapy.

José L. Blaya-Cánovas[1,2,3,#], Carmen Griñán-Lisón[2,3,4,5,#], Isabel Blancas[2,6,7], Juan A. Marchal[2,5,8,9], Cesar Ramirez-Tortosa[2,10], Araceli López-Tejada[2,3,4], Karim Benabdellah[3], Marina Cortijo-Gutiérrez[3], M.Victoria Cano-Cortés[2,3,11], Pablo Graván[2,5,12], Saúl A. Navarro-Marchal[2,5,8,12], Jaime Gómez-Morales[13], Violeta Delgado-Almenta[3], Jesús Calahorra[1,2,3], María Agudo-Lera[3], Amaia Sagarzazu[3], Carlos J. Rodríguez-González[6], Tania Gallart-Aragón[7,14], Christina Eich[15], Rosario M. Sánchez-Martin[2,3,11], Sergio Granados-Principal[2,3,4,*].

[1]UGC de Oncología Médica, Hospital Universitario de Jaén, 23007, Jaén, Spain

[2]Instituto de Investigación Biosanitaria ibs.GRANADA, University Hospitals of Granada-University of Granada, 18100, Granada, Spain

[3]GENYO, Centre for Genomics and Oncological Research, Pfizer/University of Granada/Andalusian Regional Government, 18016, Granada, Spain

[4]Department of Biochemistry and Molecular Biology 2, Faculty of Pharmacy, University of Granada, Campus de Cartuja s/n, 18071, Granada, Spain

[5]Excellence Research Unit "Modeling Nature" (MNat), University of Granada, 18100, Granada, Spain.

[6]UGC de Oncología, Hospital Universitario "San Cecilio", 18016, Granada, Spain

[7]Department of Medicine, University of Granada, 18016, Granada, Spain

[8]Biopathology and Regenerative Medicine Institute (IBIMER), Centre for Biomedical Research, (CIBM) University of Granada, 18100, Granada, Spain.

[9]Department of Human Anatomy and Embryology, Faculty of Medicine, University of Granada, 18016, Granada, Spain.

[10]UGC de Anatomía Patológica Hospital San Cecilio de Granada, 18016, Granada, Spain

[11]Department of Medicinal & Organic Chemistry and Excellence Research Unit of "Chemistry Applied to Biomedicine and the Environment", Faculty of Pharmacy, University of Granada, Campus de Cartuja s/n, 18071, Granada, Spain

[12]Department of Applied Physics, Faculty of Science, University of Granada, 18071, Granada, Spain

[13]Laboratorio de Estudios Cristalográficos IACT-CSIC-UGR, 18100, Armilla, Spain

[14]UGC de Cirugía General y del Aparato Digestivo, Hospital Universitario "San Cecilio", 18016, Granada, Spain

[15]Translational Nanobiomaterials and Imaging, Department of Radiology, Leiden University Medical Center, 2333, Leiden, the Netherlands

#These authors contributed equally

*Corresponding author: sergiogp@ugr.es

Supplementary Table S1. Characterization of PLGA NPs with docetaxel, doxorubicin, and epirubicin

Drug encapsulated	z-Average (nm)	ζ-Potential (mV)	PDI	EE (%)	LC (mg/mg)
**Docetaxel**	190.6±5.2	-16.5±0.15	0.12±0,05	80±15.52	16.05±2.61
**Doxorubicin**	165.1±1.13	-29.3±3.7	0.05±0.03	9.9±0.98	0.78±0.10
**Epirubicin**	194.3±10.8	-24.3±0.8	0.09±0.01	8.1±1.25	0.64±0.08

PDI: polydispersity index; EE: encapsulation efficiency; LC: loading capacity

Supplementary Table S2. Hematological parameters in each experimental group (n=3 mice/group)

Parameter	Control	Vehicle	PLGA (25 mg/kg)	PLGA (100 mg/kg)	NExT (25 mg/kg)	NExT (100 mg/kg)
**WBC (×10^3/µl)**	2.33±0.5	4.23±1.1	2.63±0.7	4.93±0.5	4.37±1.3	4.23±1.6
**RBC (×10^6/µl)**	7.23±0.4	8.07±0.5	6.37±2.2	7.74±0.4	7.89±0.2	7.31±0.7
**Hgb (g/dl)**	13.43±0.85	14.13±0.5	12.40±3.2	13.70±0.4	14.03±0.6	12.70±1.7
**HCT (%)**	40.9±1.95	44.43±2.2	36.03±13.7	43.07±2.1	43.87±1.6	40.73±3.6
**MCV (fL)**	56.6±0.79	55.10 ±1.9	56.17±2.1	56.25±0.9	55.63±2.2	55.67±0.7
**MCH (pg)**	18.57±0.23	17.57 ±0.6	20.17±2.9	17.40±1.1	17.80±0.7	17.30±1.0
**MCHC (g/dl)**	32.83±0.49	31.80±0.4	36.10±6.8	31.87±1.2	32.00±0.1	31.10±1.9
**PLT (×10^3/µl)**	739.67±321.9	1061.67±103.6	593.00±427.4	975.00±211.5	932.00±74.5	589.67±468.0

WBC: White Blood Cells; RBC: Red Blood Cells; Hgb: Hemoglobin; HCT: Hematocrit; MCV: Mean Corpuscular Volume; MCH: Mean Corpuscular Hemoglobin; MCHC: Mean Corpuscular Hemoglobin Concentration; PLT: Platelets
