# Supplementary material for: Autologous patient-derived exhausted nano T-cells exploit tumor immune evasion to engage an effective cancer therapy
Source: Mol Cancer. 2024 May 9;23:83. doi: 10.1186/s12943-024-01997-x (PMC11084007; doi:10.1186/s12943-024-01997-x)

Supplementary file

Autologous patient-derived exhausted nano T-cells exploit tumor immune evasion to engage an effective cancer therapy.

José L. Blaya-Cánovas[1,2,3,#], Carmen Griñán-Lisón[2,3,4,5,#], Isabel Blancas[2,6,7], Juan A. Marchal[2,5,8,9], Cesar Ramirez-Tortosa[2,10], Araceli López-Tejada[2,3,4], Karim Benabdellah[3], Marina Cortijo-Gutiérrez[3], M.Victoria Cano-Cortés[2,3,11], Pablo Graván[2,5,12], Saúl A. Navarro-Marchal[2,5,8,12], Jaime Gómez-Morales[13], Violeta Delgado-Almenta[3], Jesús Calahorra[1,2,3], María Agudo-Lera[3], Amaia Sagarzazu[3], Carlos J. Rodríguez-González[6], Tania Gallart-Aragón[7,14], Christina Eich[15], Rosario M. Sánchez-Martin[2,3,11], Sergio Granados-Principal[2,3,4,*].

Uncropped Western Blot images

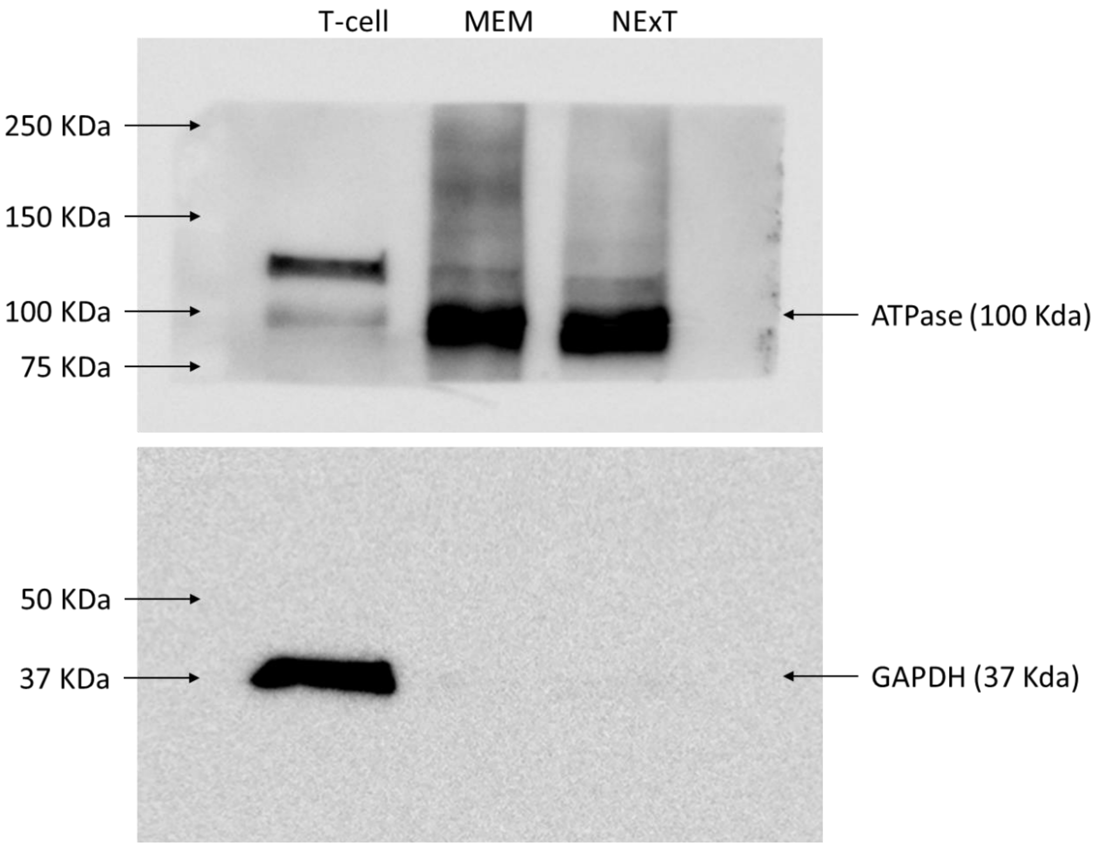

Supplement: Supplementary file 3 — Supplementary Material 3 [file 12943_2024_1997_MOESM3_ESM.pdf]
